# Iridium Stabilizes Ceramic Titanium Oxynitride Support for Oxygen Evolution Reaction

**DOI:** 10.1021/acscatal.2c04160

**Published:** 2022-11-28

**Authors:** Gorazd Koderman Podboršek, Luka Suhadolnik, Anja Lončar, Marjan Bele, Armin Hrnjić, Živa Marinko, Janez Kovač, Anton Kokalj, Lea Gašparič, Angelja Kjara Surca, Ana Rebeka Kamšek, Goran Dražić, Miran Gaberšček, Nejc Hodnik, Primož Jovanovič

**Affiliations:** † Department of Materials Chemistry, National Institute of Chemistry, Hajdrihova 19, SI-1000Ljubljana, Slovenia; ‡ Jožef Stefan International Postgraduate School, Jamova 39, SI-1000Ljubljana, Slovenia; § Department for Nanostructured Materials, Jožef Stefan Institute, Jamova 39, SI-1000Ljubljana, Slovenia; ∥ Department of Chemical and Pharmaceutical Sciences, University of Trieste, via L. Giorgieri 1, 34127Trieste, Italy; ⊥ University of Nova Gorica, Vipavska 13, SI-5000Nova Gorica, Slovenia; # Department of Surface Engineering, Jožef Stefan Institute, Jamova 39, SI-1000Ljubljana, Slovenia; ∇ Department of Physical and Organic Chemistry, Jožef Stefan Institute, Jamova 39, SI-1000Ljubljana, Slovenia; ○ Centre of Excellence for Low-Carbon Technologies, Hajdrihova 19, SI-1000Ljubljana, Slovenia; ◆ Faculty of Chemistry and Chemical Engineering, University of Ljubljana, Večna pot 113, SI-1000Ljubljana, Slovenia

**Keywords:** electrocatalysis, oxygen evolution reaction, anodic oxidation, titanium oxynitride nanotubular support, iridium nanoparticles, IL-TEM, nano lab approach, single atoms

## Abstract

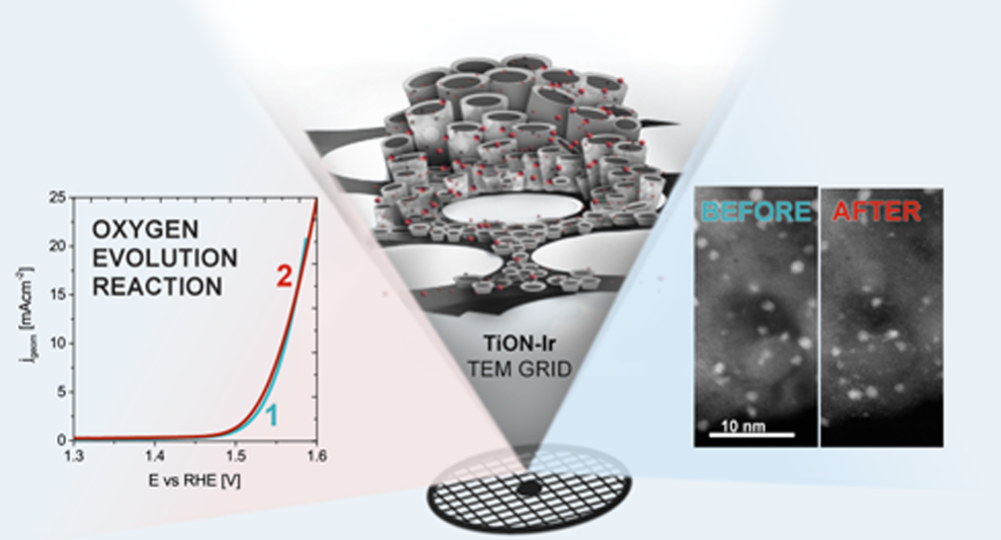

Decreasing iridium loading in the electrocatalyst presents a crucial challenge in the implementation of proton exchange membrane (PEM) electrolyzers. In this respect, fine dispersion of Ir on electrically conductive ceramic supports is a promising strategy. However, the supporting material needs to meet the demanding requirements such as structural stability and electrical conductivity under harsh oxygen evolution reaction (OER) conditions. Herein, nanotubular titanium oxynitride (TiON) is studied as a support for iridium nanoparticles. Atomically resolved structural and compositional transformations of TiON during OER were followed using a task-specific advanced characterization platform. This combined the electrochemical treatment under floating electrode configuration and identical location transmission electron microscopy (IL-TEM) analysis of an in-house-prepared Ir-TiON TEM grid. Exhaustive characterization, supported by density functional theory (DFT) calculations, demonstrates and confirms that both the Ir nanoparticles and single atoms induce a stabilizing effect on the ceramic support via marked suppression of the oxidation tendency of TiON under OER conditions.

## Introduction

Electrochemical conversion of renewable resources to electrical energy, fuels, or useful chemicals is one of the most promising directions for society to reach a clean energy landscape. Electrochemical water splitting is considered a cornerstone of such a scenario delivering hydrogen as a clean energy carrier for future power supply, energy storage matrix, and raw commodity for the chemical sector. However, further expansion of proton exchange membrane electrolyzers (PEM) as the most promising platform for sustainable hydrogen generation from intermittent renewable energy sources like solar and wind is jeopardized by its dependency on noble metal catalysts.^1^ Presently, the crucial bottleneck is the anode side of the electrolyzers, where the sluggish oxygen evolution reaction (OER) dictates the employment of expensive and scarce iridium to an unsustainable extent.^2^ Therefore, there is a strong incentive to minimize the amount of currently still irreplaceable iridium in the catalyst layer and enhance its activity and durability.^3,4^ Following the main concepts of fuel cells and platinum-based catalysts, most approaches for decreasing the loading of iridium are based on synthesizing catalyst core–shell morphologies with minimal Ir content^5−8^ by alloying iridium with other metals^7,9−13^ or by mixing iridium nanoparticles with less expensive oxides of earth-abundant elements.^14^ Especially effective is dispersing iridium nanoparticles on a high-surface-area support.^8,15−24^ However, supported OER catalysts represent a multidimensional platform encompassing many unresolved phenomena such as support electroconductivity, metal–support interactions,^25^ support morphology, and stability,^26,27^ to name a few. All of these can potentially influence electrochemical performance. Many of the relevant parameters have already been targeted for the case of titanium oxynitride support (TiON) in conjunction with the iridium catalyst. Preliminary studies have demonstrated several promising aspects of TiON-based supports for OER. Briefly, sufficiently high dispersion of iridium nanoparticles^28^ accompanied with nanotubular^29^ or nanoribbon-like support morphology^30^ and strong metal–support interactions (SMSI)^31,32^ has been documented. Therefore, further insights on resolving and understanding TiON as the OER support are justifiably needed. The fundamental questions that remain to be resolved are if and how TiON can resist oxidation toward semiconducting TiO_2_ at OER-relevant conditions. Accordingly, the underlying mechanisms and potentially also metal–support interactions^33−37^ that govern the N/O ratio in titanium oxynitride need to be resolved. To address some of these questions, we undertook a unique approach and performed careful synthesis and comprehensive characterization of the TiON-Ir system with our task-specific advanced characterization platform. We developed a proprietary procedure to anodically oxidize and thermally treat a commercial Ti TEM grid to produce TiON floating electrodes.^38^ With the subsequent deposition of Ir nanoparticles, these electrodes can then be used in a specially designed electrochemical setup, in the TEM and also other characterization tools.^39^ This way, we were able to perform electrochemical experiments and microscopic analysis on the individual grids and thus track atomically resolved local changes at identical locations. This approach is enabling a direct imaging of processes in question and not just statistical evaluation of their relevance—as is the case in the conventional ex situ TEM imaging at random positions. Herein, we show that Ir nanoparticles (Ir NPs) undergo dynamic changes leading to the formation of Ir single atoms (Ir SAs) on a TiON support. Furthermore, we demonstrate that the TiON support decorated with Ir SAs and Ir NPs is 10 times more resistant against electrochemical oxidation than its analogue without Ir. This provides a viable approach toward the stabilization and employment of ceramic-supported OER electrocatalysts.

## Results

### General Modus Operandi and Initial Structure of the Electrode

Two Ti TEM grids modified into nanotubular TiON electrodes were prepared. These, however, differed in the absence/presence of iridium nanoparticles, namely, TiON and TiON-Ir. In TiON-Ir, the TiON support is populated by iridium nanoparticles with an average particle size of 1.5 nm (Table S1). Note that this particular Ir particle size and nanotubular morphology of TiON are expected when using this particular synthesis.^29,31^ According to XPS characterization, the as-synthesized TiON contains 51 atom % of O, 23 atom % of N, and 26 atom % of Ti; for further details, see Section S2, Figure S3d–f. The excess O may be contributed to surface contamination. After the attachment of Ir nanoparticles, no significant alterations are observed from the XPS analysis in regard to TiON, whereas iridium peaks of Ir(0) metallic state (60%) and Ir oxide in the Ir(4+) state (40%) were deconvoluted (Section S2, Figure S3a–c). Subsequently, electrochemical experiments coupled with IL-TEM diagnostics were performed for each of the two analogues.

### Electrochemical Characterization Coupled with Structural Characterization

**Figure 1:**
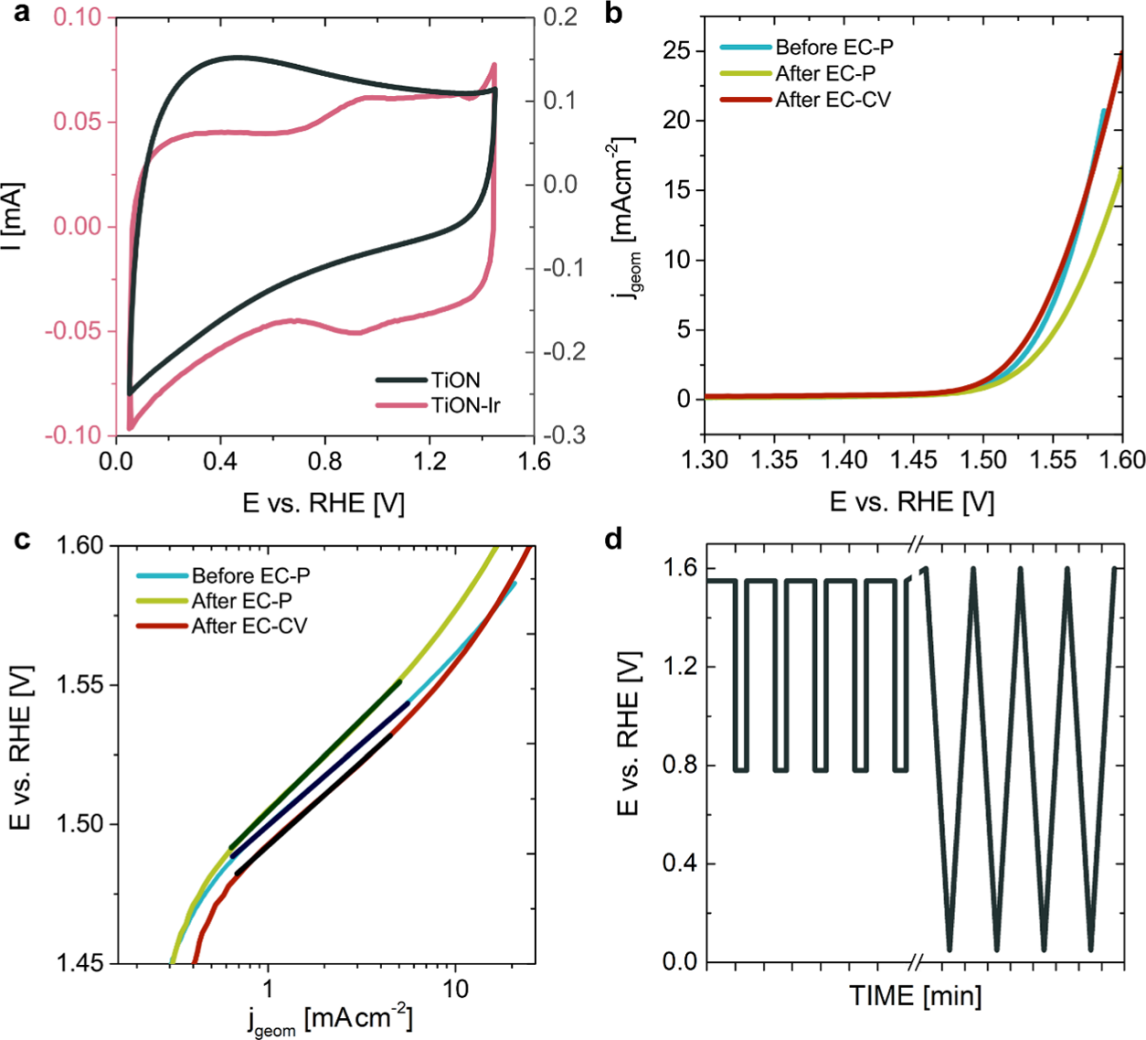
Electrochemical characterization of TiON and TiON-Ir TEM grid. (a) 100th voltammetric cycle obtained in the electrochemical pretreatment (300 mV s^–1^). (b) Geometric-area normalized OER polarization curves measured either before or after EC-P and EC-CV electrochemical biasing. (c) Tafel plots of OER polarization curves (constructed from panel (b)). (d) Potential–time diagram used for EC-P and EC-CV electrochemical biasing. Initial 10 sequences (out of 20 altogether) are shown for EC-P and 3 CVs (out of 150 altogether) for EC-CV.

The general electrochemical trends confirm that proper electric (i.e., electronic) contacting was achieved with TEM grid, and no anomalies arose from the MFE setup. We note, however, that the characteristic iridium redox behavior (Figure 1a) is somewhat different from what would typically be expected from the literature, where several redox peaks are typically observed. The deviation from the expected trend observed herein could be ascribed to the size of small iridium particles (∼1.5 nm), which seems to demonstrate steady-state growth of the oxide.^40^ Nevertheless, the corresponding OER activity (Figures S7c and 1b) agrees with the previous report on TiON-based analogues with similar Ir particle sizes.^32^ A Tafel slope of ∼60 mV dec^–1^ was determined (Figure 1c). This value is comparable to the literature values of TiON-supported Ir analogues^29,30,32^ as well as with both the bulk, rutile IrO_2_, and the electrochemically grown oxide,^22,41−43^ indicating that no significant mechanistic difference in OER with respect to other Ir-based catalysts exists.

Subsequently, two-sequence long-term electrochemical protocols (EC-P and EC-CV; described in detail in the Experimental Section) were performed and complemented with detailed statistical STEM analysis of preselected locations (i.e., identical location, IL-STEM mode). Note that the same locations were also analyzed prior to electrochemical experiments. Interestingly, according to IL-STEM analysis of the relevant structural characteristics (average particle size, nearest-neighbor distance, and circularity), no significant alterations were triggered during either of the two protocols (Table S1 and Figure S1). Nevertheless, according to the post mortem XPS analysis, both electrochemical protocols indeed altered the surface composition of iridium and TiON support. Namely, an increase in Ir(4+) and a decrease in Ir(0) were resolved in comparison to the as-synthesized state with values of 70% Ir(4+) and 30% Ir(0) after the EC-P protocol, whereas all Ir atoms transformed to the Ir(4+) oxidation state after the EC-CV protocol (Figure S3c and Table S3). This correlates with voltammetric response obtained during the two protocols (Figure S7a,b) where the voltammogram after the EC-CV perturbation is evidently wider in comparison to that after the EC-P case, indicating that more (porous) hydrous oxide is formed after the EC-CV perturbation. This is in line with the early iridium literature demonstrating that hydrous oxide predominantly forms during consequential cycling,^44−48^ whereas the fact that no metallic Ir is present after the EC-CV treatment (Table S3) might as well be supported by past works. This showed that at low potentials, the compact oxide gets reduced, while the hydrous oxide remains and can grow infinitely.^40,48^ Similarly, oxidation of the TiON support (TiON-Ir sample) after the two electrochemical protocols is clearly evident, as indicated by the N/O ratio decrease (Table S3). To decouple the influence of iridium on support oxidation, the investigation was upgraded by inspecting TiON-Ir and TiON samples independently via IL-EELS analysis, i.e., a suitable technique for analyzing light elements.

**Figure 2:**
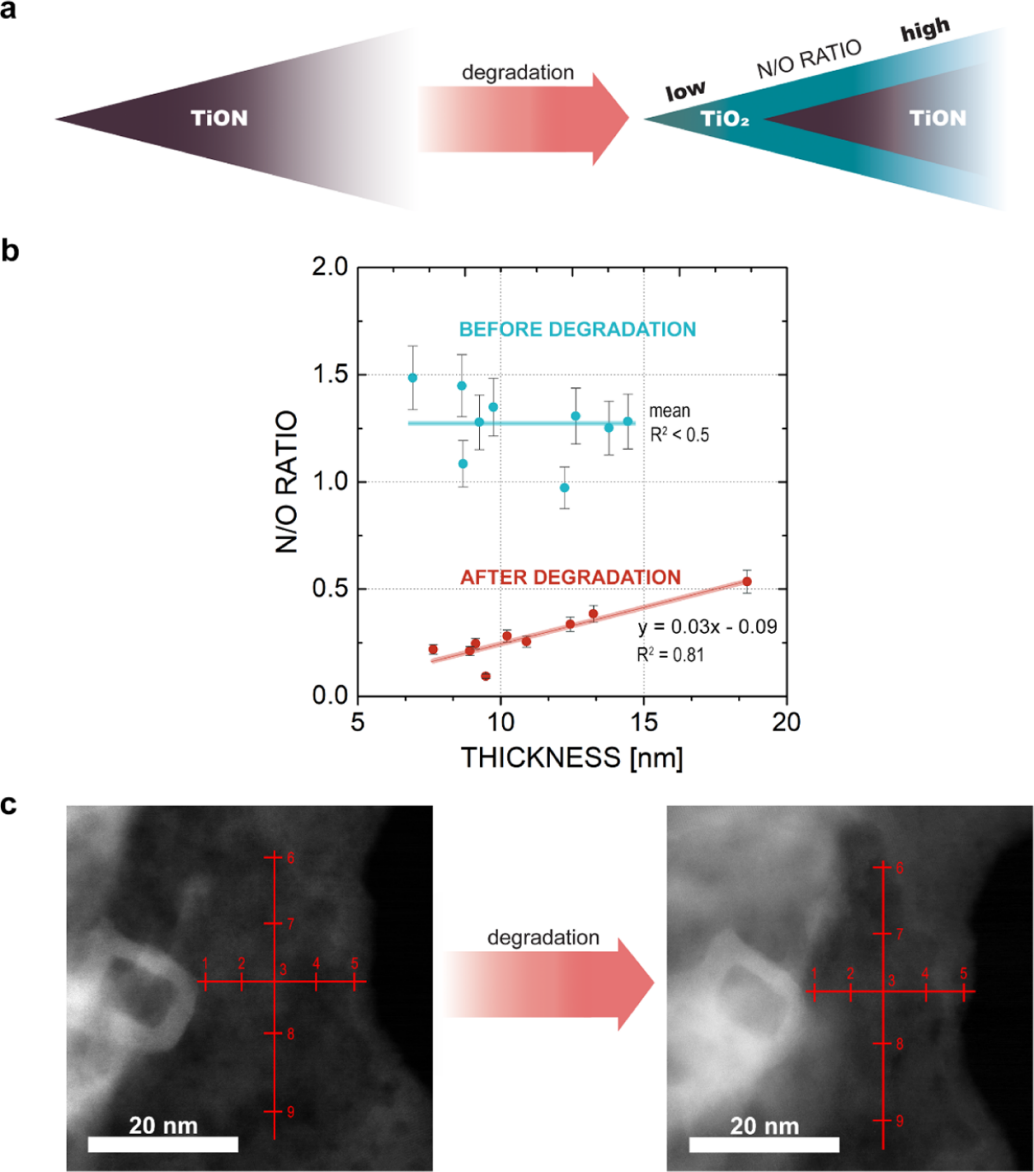
(a) Schematic of TiON support alteration during electrochemical perturbation. The average N/O ratio of the dark violet area before electrochemical perturbation is assumed to be the same as the average N/O ratio of the area after electrochemical perturbation. (b) Graphs of the N/O ratio vs thickness (nm) on the identical location of TiON sample before and after degradation. Mean value line was used instead of linear regression line because *R*^2^ < 0.5 (before degradation). (c) Identical location EELS measurement locations for TiON. The left image is the sample before electrochemical degradation, and the right image is the sample after electrochemical degradation.

**Figure 3:**
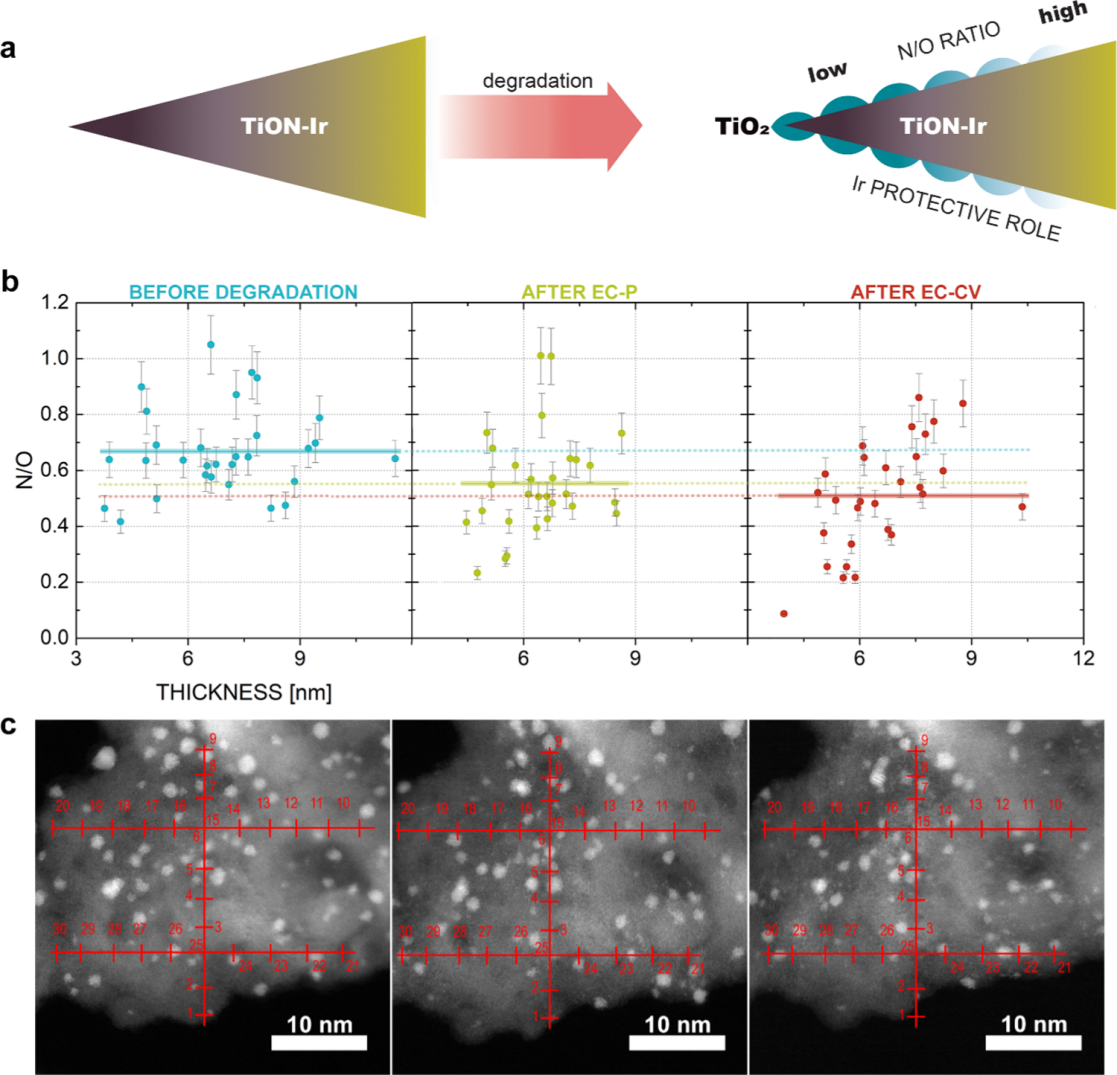
(a) Schematic of TiON-Ir support alteration during electrochemical perturbation. The average N/O ratio of the dark violet and yellow area before electrochemical perturbation is assumed to be the same as the average N/O ratio of the area after electrochemical perturbation. The protective role of Ir successfully inhibits the TiON oxidation. (b) Graph of the N/O ratio vs thickness (nm) on the identical location of TiON-Ir sample before (dark green) and after EC-P (light green) and EC-CV (red). Mean value line was used instead of linear regression line because *R*^2^ < 0.5. (c) Identical location EELS measurement locations for TiON-Ir. Before electrochemical degradation (left), after EC-P (center), and after EC-CV (right).

The initial focus of IL-EELS analysis was placed on the bare TiON analogues (locations of the measurements can be seen in Figure 2c). By comparing identical measurement points before and after electrochemical treatment, the IL-EELS analysis provides two obvious findings. The first is the evident decrease in the N/O ratio induced by the electrochemical perturbations (Figure 2b), which means that an estimated 3.0 ± 0.4 nm (mean ± standard deviation) thick TiO_2_ layer evolved (a comprehensive description of thickness estimation is provided in Section S1). The second major finding is the linear relation between the N/O ratio and the support thickness induced by electrochemistry, meaning the formed TiO_2_ layer has a constant thickness through the sample. The two findings provide a firm evidence that the electrochemical oxidation of TiON to a TiO_2_ layer is limited to the surface/near-surface region only, whereas the TiON core is left intact (Figure 2a). By contrast, IL-EELS analysis of the TiON-Ir sample provides different findings. First, in this case, the N/O ratio decreases only slightly during electrochemistry (i.e., an estimated TiO_2_ layer thickness of 0.3 ± 0.3 nm after EC-P and 0.0 ± 0.6 nm after the EC-CV protocol, see Section S1), indicating that Ir induces a 10-fold (10 ± 3) thinner oxide layer and thus efficient resistance of TiON toward oxidation as schematically depicted in Figure 3a. Second, no clear relationship exists between the N/O ratio and support thickness in the case of TiON-Ir regardless of whether the sample was electrochemically treated or not (Figure 3b). These two deviations from the bare TiON analogues prove that iridium significantly impacts TiON oxidation (for measurement locations, see Figure 3c).

**Figure 4:**
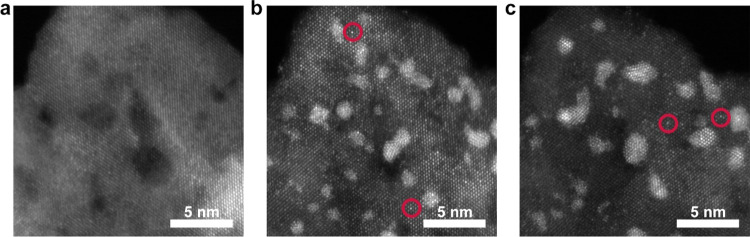
STEM-HAADF identical location images of (a) TiON, (b) TiON-Ir after EC-P, and (c) TiON-Ir after EC-CV. An identical location image of TiON-Ir before degradation was not taken. Examples of Ir single atoms occupying Ti regular sites are encircled in red.

For a deeper understanding of the observed phenomena, atomically resolved IL-TEM analysis for both analogues was performed—with a strong focus on TiON-Ir. Various structural transformations resulting from the electrochemical operation can be resolved (Figure S4), whereas OER performance (normalized per geometric surface area and extracted at 1.55 V) initially declines but subsequently increases again (Figure 1b,c). The performance trend should be ascribed to the nature of electrochemically formed Ir oxide, which depends on the perturbation mode and is further discussed in Section S4. From a structural perspective, one particular phenomenon stands out, i.e., the single atoms (Ir SAs), which appeared during the electrochemical treatment (Figure 4). Ir SAs can be seen in Figure 4b,c as brighter spots in the crystal lattice (some are encircled in red). A detailed inspection of the zone axis reveals that Ir SAs tend to occupy Ti regular sites on the surface of the crystallized TiON support. The Ir SAs’ abundance trend seems to be related to the perturbation mode, i.e., there are more Ir SAs after EC-CV (0.53 nm^–2^) compared to those after EC-P (0.29 nm^–2^) (Figure S2c,d). This seems to be in line with the formation of more hydrous oxide during the EC-CV case (Figure S7a,b) and implies that a transient electrochemical perturbation to low cathodic potentials accelerates SA formation. However, to comprehensively correlate the amount of Ir SAs with specific electrochemical biasing, more focused studies are needed. Nevertheless, the SA trend is inversely proportional to the TiO_2_ thickness where a higher Ir SA amount coincides with a smaller TiO_2_ thickness (0.0 ± 0.6 nm after EC-CV and 0.3 ± 0.3 nm after EC-P protocol). Interestingly, post mortem Raman analysis is also in line with this rationale, showing that the highest support stability under increasing laser power is reached after EC-CV perturbation (see Section S3). This further implies that the presence of Ir SAs plays an important role in inhibiting TiON oxidation. We emphasize that care was taken to be certain that the appearance of Ir SAs was indeed of electrochemical origin, which was confirmed by analyzing the identical location before Ir NP attachment. Note that no such spots are present on the TiON support (Figures 4b and S2a), and only a few (0.02 nm^–2^) are present after Ir NP attachment (Figure S2b). We note, however, that for the case of Ir NP, TiON oxidation under laser power is in fact promoted (see Section S3); therefore, correlating the stability behavior under the two regimes (i.e., electrochemical and Raman laser) should be taken with great caution. To rationalize the observed TiON oxidation trend, a theoretical investigation was performed as reported in continuation.

### DFT Characterization of the Role of Ir in Stabilizing TiON

**Figure 5:**
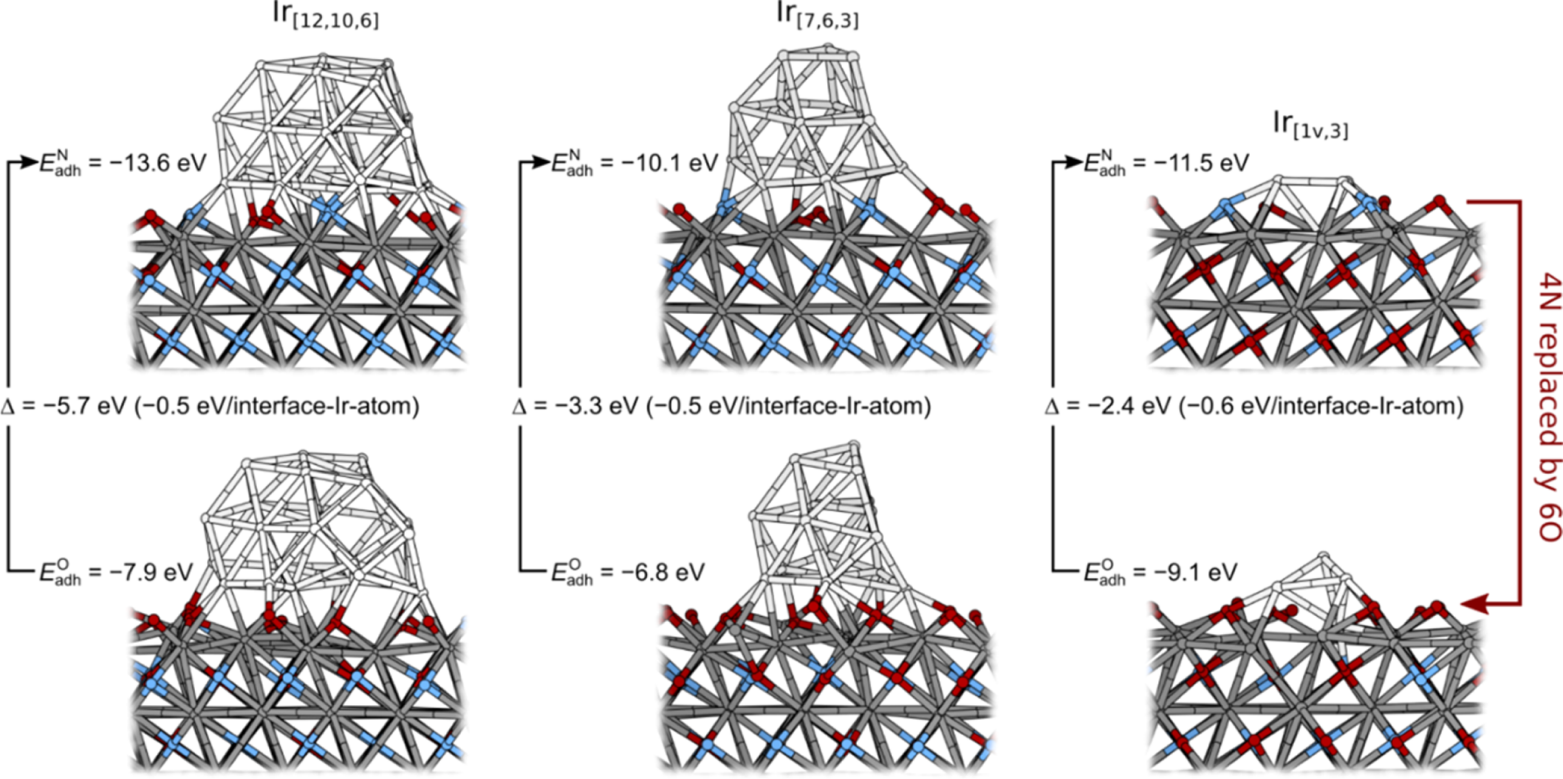
Snapshots of Ir_[12,10,3]_, Ir_[7,6,3]_, and Ir_[1v,3]_ NPs on the TiON(111) surface of Ti-vac_O_ type (Figure S9). The top row shows NPs on “pristine” TiON(111), whereas the bottom row shows the corresponding structures with 4 N ions below the NP replaced by 6 O ions. The adhesion energies and the resulting Ir-induced N preferences, Δ of eq 2, are reported (Δ values normalized to a single interface Ir atom are also given). The Ir_[*i*,*j*,*k*]_ denotation indicates a three-layer NP consisting of *i*, *j*, and *k* Ir atoms in the bottom, middle, and top layers. The Ir_[1v,3]_ consists of one Ir atom incorporated into a surface vacancy and three Ir atoms above it.

In the previous publication,^31^ we showed that the adhesion of Ir NPs on the TiON support is considerably enhanced by N atoms, i.e., by replacing N atoms, located below NPs, with O atoms, the adhesion of Ir NPs is considerably weakened. As shown in Figure 5, this is true even if N atoms are replaced by O atoms stoichiometrically, meaning that 2*n* N atoms are replaced by 3*n* O atoms, where *n* ≥ 1 (*n* = 2 for all of the cases shown in Figure 5). Conversely, this implies that Ir NPs stabilize the TiON structure in the sense that the driving force for replacing N atoms with O atoms is diminished when Ir NPs are present. Indeed, the “Ir-induced N preference”, calculated by eq 2, is sizeable and ranges from −5.7 eV (for the largest considered Ir_[12,10,6]_ NP) to −2.4 eV (for the smallest considered Ir_[1v,3]_ NP). The three considered NPs have 10, 7, and 4 Ir atoms in contact with the TiON support, and renormalizing the Ir-induced N preference to an interface Ir atom gives the values within −0.5 and −0.6 eV per interface Ir atom.

**Figure 6:**
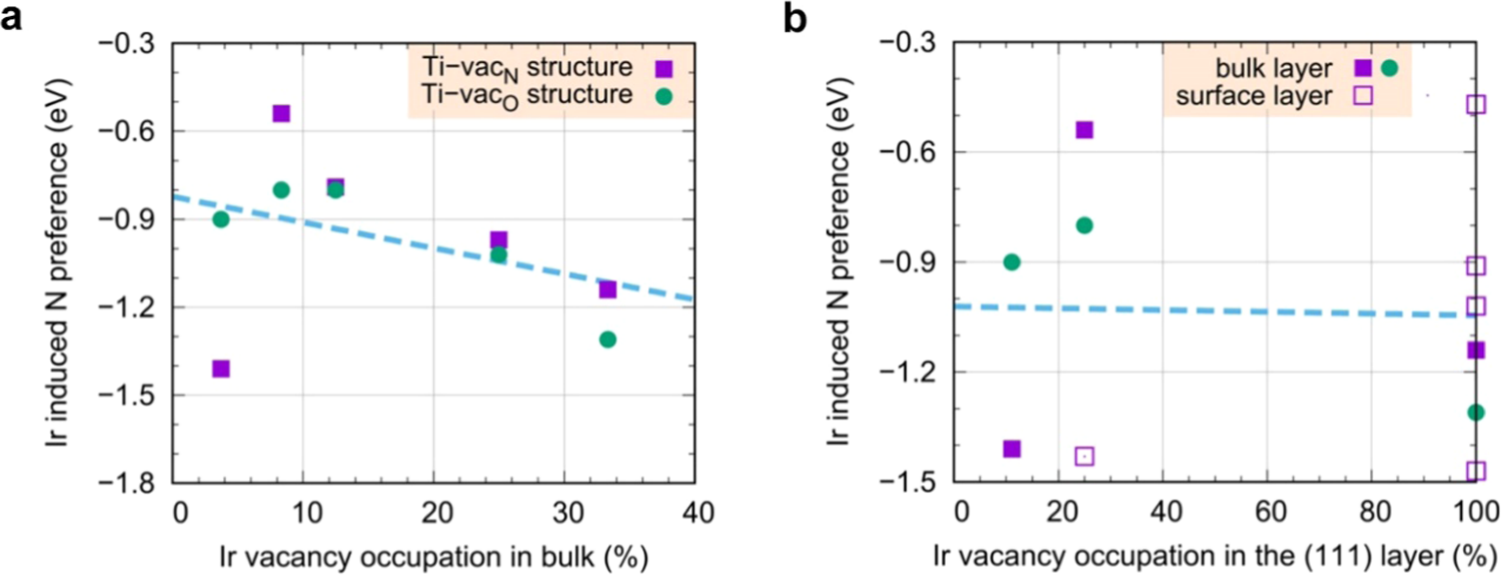
Ir-induced N preference, Δ_1:1_ of eq 4, as a function of Ir single atom concentration. A bulk concentration of 100% implies that all Ti vacancies are occupied by Ir atoms, whereas a “surface” concentration of 100% implies that Ir atoms occupy all Ti vacancies in a given Ti(111) layer. (a) Δ_1:1_ as a function of bulk Ir concentration for various considered bulk Ir/TiON structures (both Ti-vac_O_- and Ti-vac_N_-type structures are considered, Figure S9). (b) Δ_1:1_ as a function of Ir concentration in the (111) layer for various considered surface (open symbols) and bulk (solid symbols) Ir/TiON structures (purple squares and green points refer to Ti-vac_N_ and Ti-vac_O_ structures, respectively). The blue dashed lines guide the eye and indicate the Δ_1:1_ average (the lines are linear fits to data points). For (111)-rotated bulk structures, the (111)-layer concentration of 100% corresponds to a bulk concentration of 33% because TiON(111) consists of A–B–C stacking of Ti layers, and in the corresponding calculations, Ir atoms are located in only one of the three Ti layers.

Furthermore, calculations indicate that single Ir atoms can incorporate themselves into Ti vacancies in the TiON structure. Such Ir single atoms, either on the surface or in bulk, also stabilize the TiON structure because they reduce the driving force for replacing N atoms with O atoms. This effect is quantified through “Ir-induced N preference” of eq 4 in Figure 6, where Ir single atoms, incorporated into Ti vacancies in bulk and on the surface, are considered at various concentrations (the corresponding bulk and surface structures are shown in Figures S11 and S12). While various considered cases display different Ir-induced N preferences, it seems that stabilization induced by a single Ir atom is about 1 eV per N atom on average. That is, by replacing a single N atom, bonded to Ir, with an O atom, the binding (adhesion) of Ir single atom in a TiON bulk and on a TiON(111) surface reduces by about 1 eV.

To summarize, calculations indicate that the Ir-induced N preference of Ir NPs is about −0.5 eV per interface Ir atom. For Ir single atoms, the Ir-induced N preference of an Ir atom is about −1 eV per N atom. Both findings are in line with support oxidation trends obtained via IL-EELS analysis.

## Conclusions

In summary, we carried out a comparative investigation of bare TiON support and its OER analogue, i.e., the Ir-TiON-supported electrocatalyst. To ensure comparable conditions for a detailed structural characterization, both analogues were synthesized directly on a Ti TEM grid and characterized via IL-TEM after the sequential electrochemical treatment in a modified floating electrochemical cell configuration. Fascinatingly, the electrochemically evolved TiO_2_ layer on the bare TiON support was estimated to be 10 times (10 ± 3) thicker than in the case of TiON-Ir analogue according to IL-EELS measurements, exposing the instrumental influence of Ir on support oxidation. Atomically resolved IL-STEM analysis revealed the presence of single Ir atoms, which can be directly related to electrochemical perturbation. Furthermore, the electrochemically formed Ir SAs were located in Ti regular sites and proposed to be associated with the increased stability of the TiON support. This hypothesis was supported by DFT calculations, which showed that both Ir NPs and SAs contributed to the improved electrochemical stability of TiON. In particular, the presence of Ir reduces the driving force for the replacement of N by O. Accordingly, the results presented herein offer a credible strategy for stabilization and further implementation of ceramic-based OER catalysts.

## Experimental Section

### Synthesis of the TiON-Ir Catalyst

**Figure 7:**
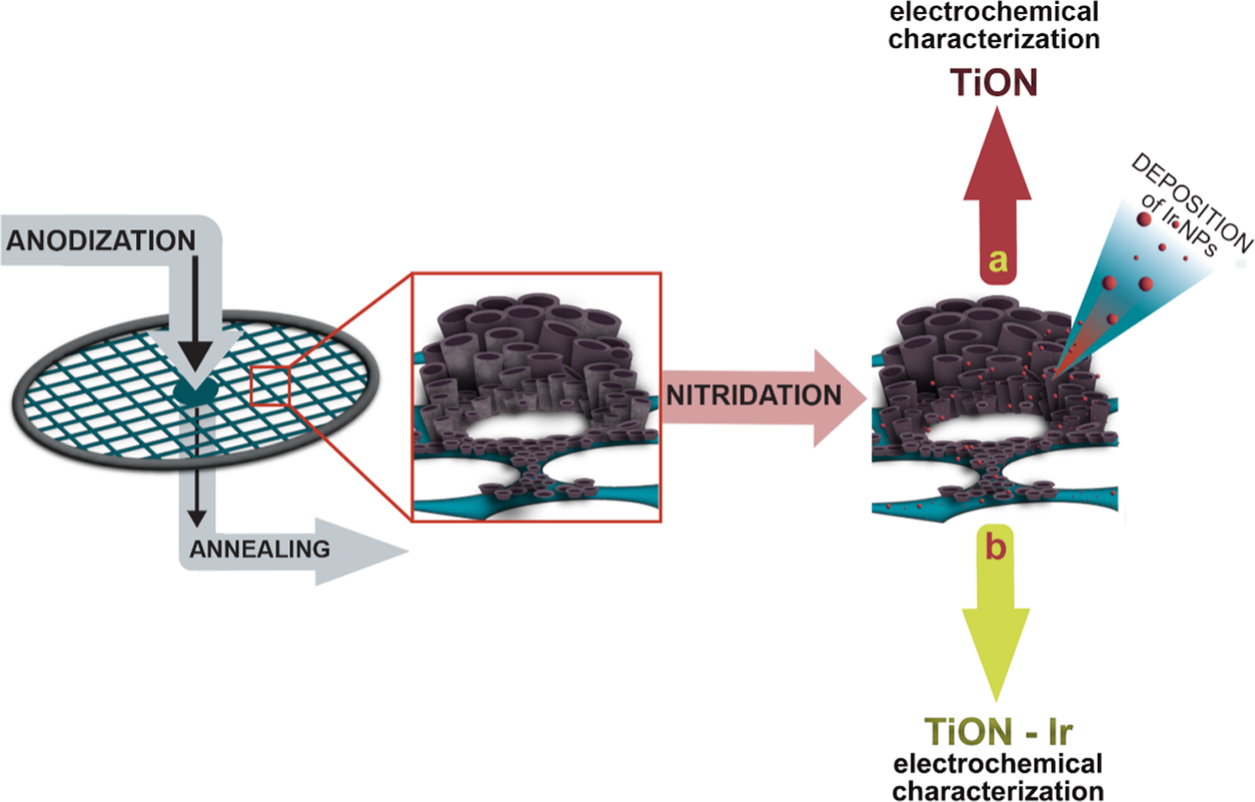
Synthesis procedure for (a) TiON and (b) TiON-Ir samples.

The TiON-Ir catalyst was prepared following the procedure shown in Figure 7b. In the first step, a Ti TEM grid (3.05 mm diameter, 400 mesh, SPI Supplies) was subjected to potentiostatic anodization in a two-electrode electrochemical cell using a stainless steel counter electrode and an anodization electrolyte consisting of 0.3 wt % NH_4_F (99.99%, Sigma-Aldrich) and 2 vol % deionized water in ethylene glycol (99.5%, Carlo Erba Reagents). The anodizing voltage was kept constant at 40 V and the anodizing time was 30 min. The anodization of a very small titanium grid was enabled by developing an anodization apparatus into which the grid was inserted, sealed, and connected to electrical contact.^38^ The procedure resulted in an amorphous TiO_2_ nanotube film, which was then washed with deionized water and dried with nitrogen. The anodized grid was annealed at 450 °C for 1 h in air to convert the amorphous TiO_2_ into an anatase structure. Afterward, a second annealing was performed in an ammonia atmosphere at 700 °C for 2 h to convert the crystalline TiO_2_ nanotube arrays into the TiON substrate. The flow of pure ammonia gas was kept constant at 50 cm^3^ min^–1^ and a pressure of 1 atm. In the last step, iridium nanoparticles were deposited on the TiON substrate, and to this end, the following procedure was developed. First, 15 mg of iridium(III) bromide hydrate (Sigma-Aldrich, St. Louis, MO) was dissolved in 1.5 mL of water at 50 °C. Then, the solution was dip-coated on the TiON substrate with a withdrawal speed of 1 cm s^–1^ and dried at 50 °C. Afterward, the sample was thermally treated in a 5% H_2_/Ar mixture. The temperature was increased at a rate of 2 °C min^–1^ to 400 °C and held for 1 h, with the subsequent cooling rate to room temperature being 3 °C min^–1^. The TiON support analogue without Ir was prepared the same way, however, omitting the last step (deposition of Ir nanoparticles) as shown in Figure 7a.

### Materials Characterization

#### Scanning Transmission Electron Microscopy

IL-STEM imaging was performed in a Cs-corrected scanning transmission electron microscope (Jeol ARM 200 CF) equipped with a Jeol Centurio SDD EDX spectrometer and GATAN Quantum ER dual-electron energy loss spectroscopy (EELS). An operational voltage of 80 kV was employed. The images were taken in STEM mode (BF and HAADF). Both STEM and EELS analyses were done at a probe size of 6 C and 3 cm effective camera length.

#### Statistical Analysis of Ir Nanoparticles

Segmentation of Ir nanoparticles on images was done using a custom-made algorithm based on adaptive thresholding. Discrepancies from the ground truth were corrected manually. The information about their area, nearest-neighbor distance, and circularity was collected with ImageJ (Fiji distribution).^49^ Statistics were done in Excel.

#### Raman Spectra

Raman spectra of samples were recorded using a confocal WITec α 300 Raman spectrometer. The laser excitation wavelength was 532 nm and the integration time was 2 s. As-measured spectra are shown in the spectral range of 20–1100 cm^–1^ in Figure S5. However, to make their comparison easier, we divided these spectra by various factors, down to a similar size shown in Figure S6. All samples were measured using a special protocol that can serve as a sample stability estimation. Specifically, Raman spectra were recorded sequentially at a particular site using increasing laser powers of 0.6, 1.4, 3.4, 7.3, and 13.5 mW. Each sample was examined on three sites. The samples suffered degradation at higher laser powers, but the extent of degradation can be taken as an estimation of their stability.

#### X-ray Photoelectron Spectroscopy

X-ray photoelectron spectroscopy (XPS) was used to analyze the surface composition and chemical status of grid samples with TiON-Ir catalysts using a PHI-TFA XPS spectrometer produced by Physical Electronics Inc. and equipped with an Al-monochromatic source. The analysis depth with XPS is about 3–5 nm, and the analysis area was 0.4 mm in diameter. Two measurements on every sample were performed. Surface composition was calculated not considering carbon, supposing it originates from surface contamination.

#### Electrochemical Measurements

**Figure 8:**
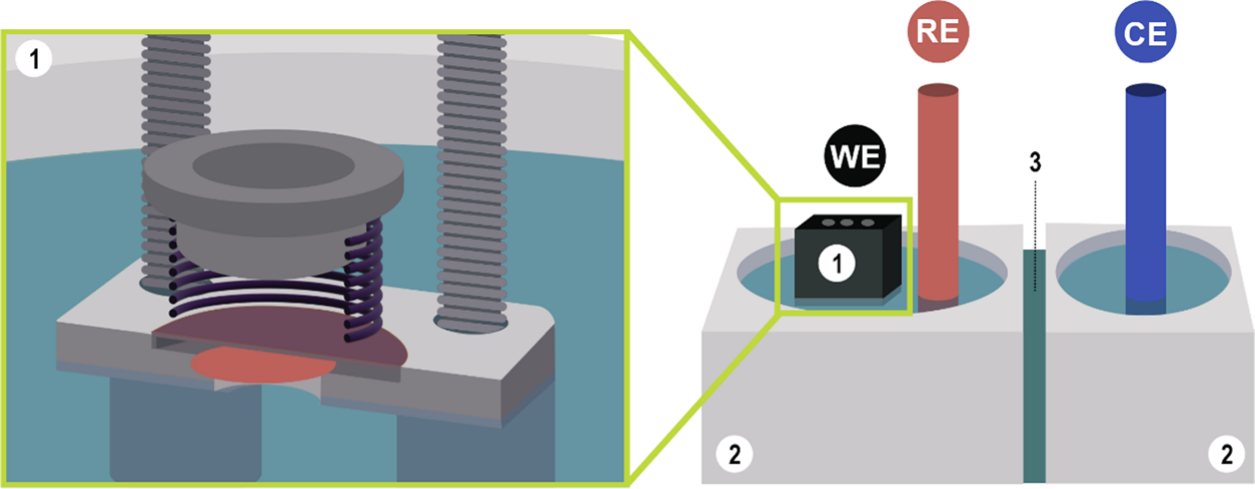
Schematic presentation of modified floating electrode setup: (2,3) general overview of the system and (1) detailed cross section of the modified floating electrode.

A recently introduced modified floating electrode (MFE) apparatus was employed to conduct electrochemical measurements.^50^ This novel approach allows performing the electrochemical experiment on a TEM grid, which serves as a working electrode (Figure 8). The floating compartment consists of a two-piece Teflon housing assembled with Tekka Peek screws. Between these elements, a Ti TEM grid working electrode, gas diffusion layer (GDL, 280 μm thickness) with 40% Teflon weight wet proofing (Toray Carbon paper 090, Fuel Cell Store), and two metallic cones with a spring between them are placed on top of each other. GDL with hydrophobic properties serves as a separator between the electrolyte and metallic cones and spring, used as electric contacts of the working electrode. All electrochemical experiments were performed in a two-compartment Teflon cell (H-cell). Floating and reversible hydrogen reference (HydroFlex, Gaskatel) electrodes were placed in the first compartment, and a Pt mesh (GoodFellow 50 × 50 mm) counter electrode was placed in the second compartment (Figure 8). The compartments were separated with a Nafion membrane (Nafion 117, Fuel Cell Store). Electrochemical experiments coupled with IL-TEM diagnostics were performed, one for each of the two analogues, i.e., TiON-Ir or TiON grid. At first, proper electrochemical contacting was assessed with a voltammetric pretreatment (300 mV s^–1^, 0.05–1.45 V, Figure 1a) to identify typical electrochemical characteristics of iridium in the TiON-Ir sample. Subsequently, electrocatalytic performance was determined by linear sweep voltammetry (LSV) measurement (20 mV s^–1^). The OER polarization curve was normalized either per geometric surface area or per iridium surface charge. In the latter, the characteristic Ir(III/IV) redox peak between 0.6 and 1.1 V was integrated, as this charge is correlated to the number of active sites.^51−53^ In this way, the contribution of the capacitive current originating from the TiON support is roughly eliminated.^54^ In continuation, a degradation treatment was conducted, i.e., twenty 5 min potentiostatic intervals at 1.55 V were interrupted with 2 min steps at 0.78 V (hereafter referred to as EC-P, Figure 1d). We emphasize that this particular protocol was selected based on our preliminary work. Effective bubble management is instrumental for adequate exploitation of the identical location approach since subsequent structural characterization of preselected identical locations (IL-TEM, IL-EELS) would be misleading in its absence. For example, if a specific location is subjected to bubble coverage, it will not restructure under electrochemical perturbation. After the EC-P protocol, an even more rigorous electrochemical protocol was employed to intentionally perturb the structure of Ir NPs and thus induce some visible changes in the TEM micrographs. Here, the potential window was widened (150 cycles, from 0.05 to 1.6 V, referred to as EC-CV, Figure 1d) to enhance the oxidation and reduction reactions of Ir and subsequently also Ir dissolution.^55−57^

In the case of the TiON sample, a potentiostatic treatment of 1.6 V for 30 min was selected as a degradation treatment. We emphasize that in this particular case, potentiostatic conditions are not problematic in terms of oxygen bubble management as no Faradaic response under OER-relevant potentials was observed^43^ (data not shown). Nevertheless, a separate TiON-Ir sample was prepared, electrochemically treated under identical conditions as the TiON sample (1.6 V for 30 min) and analyzed with TEM, the results of which are consistent with EC-P and EC-CV cases (see Section S5). The ohmic resistance between the working and reference electrode was measured using the high-frequency intercept of an impedance scan (measured at an open-circuit potential). Ohmic drop compensation (85% was compensated for) was applied during electrochemical experiments via the positive feedback mode. The uncompensated resistance value varied between 3 and 4 Ω.

#### DFT Calculations

Calculations were performed with the PWscf code from Quantum ESPRESSO^58^ using the GGA + *U* method^59,60^ with the exchange-correlation functional of Perdew–Burke–Ernzerhof (PBE).^61^ Kohn–Sham orbitals were expanded in a plane-wave basis set with a kinetic energy cutoff of 50 Ry (575 Ry for the charge density). We used the projector-augmented-wave (PAW) potentials^62^ obtained from pslibrary.^63−65^ The *U* parameter for Ti ions was calculated self-consistently from a TiON bulk structure using the hp.x code that utilizes the density functional perturbation theory scheme.^66^ The model of the TiON bulk was taken from our previous publication;^31^ it consists of a rock-salt crystal structure with two interpenetrating fcc lattices of O/N anions and Ti cations with 25% of Ti vacancies. There are seven atoms (3Ti, 2N, 2O) and 1 Ti-vacancy in the unit cell; the stoichiometry of this TiON bulk model is thus Ti_3_O_2_N_2_ (or Ti_1.5_ON). The cubic unit cell of the TiON bulk can be seen as consisting of a TiO(001) layer and a TiN(001) layer, hence a Ti-vacancy can be located in either of the two layers (Figure S9). The two resulting “ordered” structures, based on the unit cell with Ti-vacancy in either the TiO(001) or the TiN(001) layer, are labeled as Ti-vac_O_ and Ti-vac_N_, respectively. Ti-vac_O_ and Ti-vac_N_ structures display an almost identical calculated lattice parameter of 4.17 Å and a similar self-consistent value of the effective *U* parameter, 4.0 and 3.9 eV, respectively. Brillouin zone integrations were performed with the Methfessel–Paxton smearing^67^ of 0.01 Ry and a uniformly shifted 4 × 4 × 4 *k*-mesh for the cubic unit cell of the TiON bulk. For other calculations, *k*-grids were of comparable quality except for Ir nanoparticles on the TiON support where calculations were performed with only the Γ *k*-point.

Experiments revealed^31^ that the majority surface of the TiON substrate is (111), hence we modeled the (111) surface with symmetric nonpolar slabs consisting of five Ti layers terminated by an O/N layer on both sides of the slab (Figure S10): a majority of calculations were performed with stoichiometric slabs that contain only 50% of O and N ions in the surface O/N layer, while a minority of calculations were also performed with slabs that contain 100% of the O and N ions in the surface O/N layer.

The adhesion energy of Ir nanoparticles (NPs) on the TiON support was calculated as
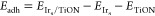
1where *E*_Ir*_n_*/TiON_, *E*_Ir_*n*__, and *E*_TiON_ are total (potential) energies of the relaxed Ir_*n*_/TiON(111) system, relaxed standalone Ir_*n*_ nanoparticle, and relaxed bare TiON(111) slab, respectively.

We utilized the following strategy to address if Ir NPs diminish the driving force for replacing N atoms with O atoms. First, we calculated the adhesion energy of an Ir NP on a “pristine” TiON(111) surface (*E*_adh_^N^) and then on the surface, where 2*n* N atoms below the NP were “stoichiometrically” replaced by 3*n* O atoms (*E*_adh_^O^). The “Ir-induced N preference” was then calculated as
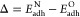
2With this definition, negative values of Δ indicate that Ir NPs stabilize the “pristine” TiON structure against replacement of N atoms by O atoms and vice versa for positive values of Δ.

Similar to Ir NPs, the Ir-induced N preference was also calculated for Ir single atoms. The binding (or adhesion) energy of an Ir atom in a Ti-vacancy is calculated analogously to eq 1, i.e.
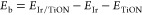
3where the energy terms have a similar meaning as in eq 1, except that an Ir single atom is considered here, and TiON can also refer to bulk structures. The Ir-induced N preference was then calculated as the difference between the pristine *E*_b_^N^ and the O-replaced *E*_b_^O^. For the latter, a single N atom was replaced by an O atom (i.e., the replacement of N by O was done in a 1:1 ratio). The Ir-induced N preference was then calculated as
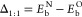
4where the subscript 1:1 indicates that a single O atom was replaced by one N atom; that is, Δ_1:1_ measures the Ir-induced N preference of a single Ir atom per replaced single N atom.

## Supporting Information Available

Results of statistical IL-STEM-HAADF analysis, detailed explanation of TiO_2_ layer thickness determination, XPS characterization description, Raman spectroscopy characterization description, iridium redox charge-normalized OER performance, EELS analysis data after potentiostatic treatment, and structures used in DFT calculations (PDF)
