# Supplementary material for: Iridium Stabilizes Ceramic Titanium Oxynitride Support for Oxygen Evolution Reaction
Source: ACS Catal. 2022 Nov 28;12(24):15135–45. doi: 10.1021/acscatal.2c04160 (PMC9764282; doi:10.1021/acscatal.2c04160)
Supplement: Supplementary file 1 — cs2c04160_si_001.pdf [file cs2c04160_si_001.pdf]

SUPPORTING INFORMATION

Iridium Stabilizes Ceramic Titanium Oxynitride Support for Oxygen Evolution Reaction

Gorazd Koderman Podboršek,[1,2,∇] Luka Suhadolnik,[3,4 ∇,*] Anja Lončar,[1,5] Marjan Bele,[1,*] Armin Hrnjić,[1,5] Živa Marinko,[2,3] Janez Kovač,[6] Anton Kokalj,[2,7] Lea Gašparič,[2,7,8] Angelja Kjara Surca,[1] Ana Rebeka Kamšek,[1,9] Goran Dražić,[1,2] Miran Gaberšček,[1] Nejc Hodnik,[1,2] Primož Jovanovič[1,*]

[1] *Department of Materials Chemistry, National Institute of Chemistry, Hajdrihova 19, SI-1000 Ljubljana, Slovenia*

[2] *Jožef Stefan International Postgraduate School, Jamova 39, SI-1000 Ljubljana, Slovenia*

[3] *Department for Nanostructured Materials, Jožef Stefan Institute, Jamova 39, SI-1000 Ljubljana, Slovenia*

[4] *Department of Chemical and Pharmaceutical Sciences, University of Trieste, via L. Giorgieri 1, 34127 Trieste, Italy*

[5] *University of Nova Gorica, Vipavska 13, SI-5000 Nova Gorica, Slovenia*

[6] *Department of Surface Engineering, Jožef Stefan Institute, Jamova 39, SI-1000 Ljubljana, Slovenia*

[7] *Department of Physical and Organic Chemistry, Jožef Stefan Institute, Jamova 39, SI-1000 Ljubljana, Slovenia*

[8] *Centre of Excellence for Low-Carbon Technologies, Hajdrihova 19, Ljubljana, Slovenia*

[9] *Faculty of Chemistry and Chemical Engineering, University of Ljubljana, Večna pot 113, 1000 Ljubljana*

[∇] Authors contributed equally

* Corresponding authors: Dr. Luka Suhadolnik, e-mail: luka.suhadolnik@ijs.si

Dr. Marjan Bele, e-mail: marjan.bele@ki.si

Dr. Primož Jovanovič, e-mail: primoz.jovanovic@ki.si

Table S1: Table showing changes of statistical values obtained from IL-STEM-HAADF images before and after EC-P and after EC-CV of TiON-Ir sample. Values are written as mean ± standard deviation.

TiON-Ir DEGRADATION			
	BEFORE	AFTER	
		EC-P	EC-CV
**Number of particles**	1709	1698	1573
**Particle size (nm^2)**	1.53 ± 1.43	1.53 ± 1.38	1.55 ± 1.56
**Nearest neighbor distance (nm)**	0.43 ± 0.23	0.44 ± 0.24	0.45 ± 0.24
**Circularity**	0.84 ± 0.08	0.83 ± 0.09	0.82 ± 0.09

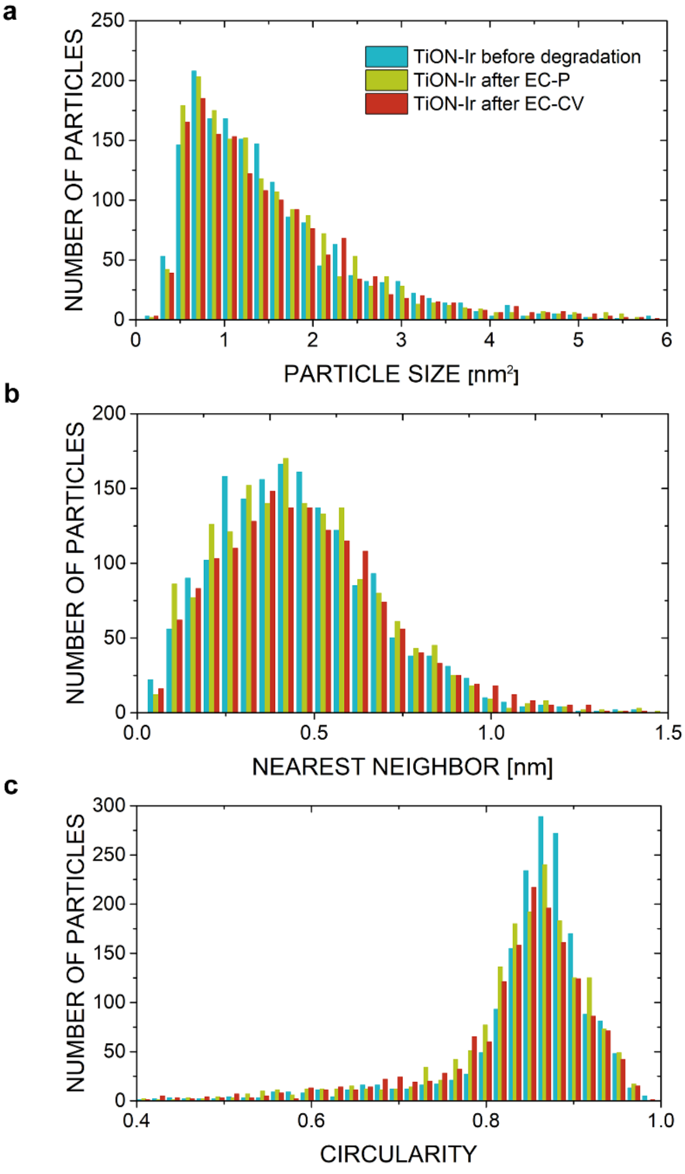

Figure S1: Statistical analysis of Ir nanoparticles before and after EC-P and after EC-CV for TiON-Ir sample. **a)** Particle size distribution, **b)** particle dispersion and **c)** particle circularity.

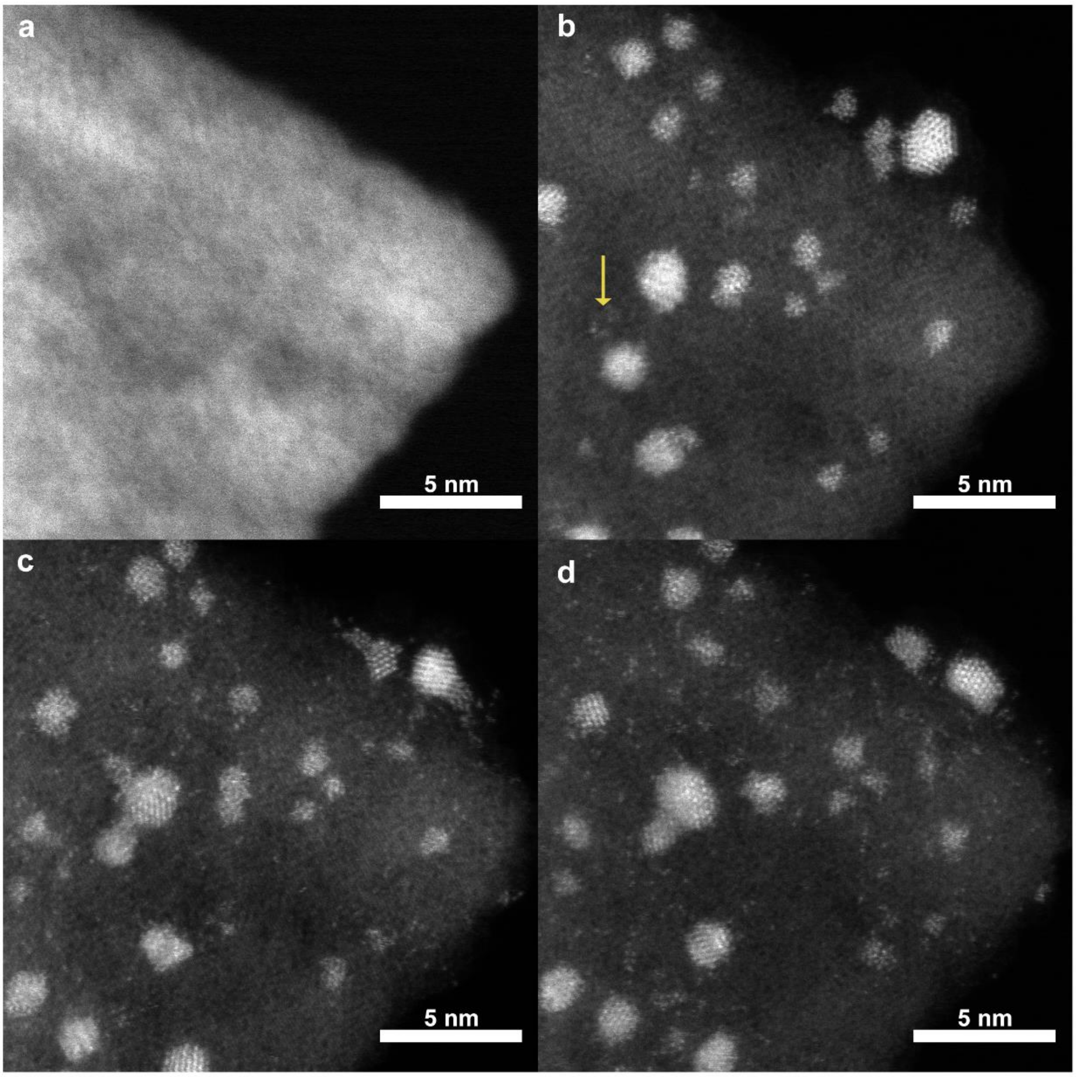

Figure S2: STEM-HAADF images of TiON-Ir sample after **a)** nitridation, **b)** addition of Ir, **c)** EC-P, and **d)** EC-CV.

S1. Estimation of TiO_2 layer thickness after degradation of TiON and TiON-Ir

One potentially viable way of estimating the TiO_2 layer thickness evolved under electrochemical conditions is from a linear regression extrapolation. If we extrapolate the linear regression formula of TiON after the electrochemical perturbation (**Figure 2** after degradation equation) to (N/O ratio)=0, we get a value of thk(TiO_2)=2.8 nm, where thk(TiO_2) represents the thickness of TiO_2 layer without nitrogen. However, one cannot use this method to estimate the thickness of TiO_2 layer for the TiON-Ir analogues after electrochemical perturbation because

of the low R^2 value of the linear regression model for N/O ratio vs. thickness (0.0143, 0.0598, 0.3404, **Figure 3**). Hence, an alternative approach is needed for TiON-Ir that can also be used for TiON to compare both results. Initially, one can assume that the two average N/O ratios of TiON and TiON core (before and after electrochemical perturbation, respectively) are the same (**Figure 2a**). Then, one asks themselves, how thick does the TiO_2 layer after the electrochemical perturbation need to be in order to obtain the same N/O ratio as in every data point in the graph in **Figure 2** (after degradation data points). This estimation method assumes the formation of a pure TiO_2 layer as the only oxidation mechanism.

$$\mathrm{N/O\ ratio\ before\ degradation} = (\mathrm{N/O})_\mathrm{b} = \frac{n(\mathrm{N})_\mathrm{b}}{n(\mathrm{O})_\mathrm{b}} \quad \text{(S1)}$$

$$\mathrm{N/O\ ratio\ after\ degradation} = (\mathrm{N/O})_\mathrm{a} = \frac{n(\mathrm{N})_\mathrm{a}}{n(\mathrm{O})_\mathrm{a}} \quad \text{(S2)}$$

$$(\mathrm{N/O})_\mathrm{a} = \frac{n(\mathrm{N})_\mathrm{a}}{n(\mathrm{O})_\mathrm{a}} = \frac{n(\mathrm{N})_\mathrm{b}}{n(\mathrm{O})_\mathrm{b} + n(\mathrm{O})_{\mathrm{TiO_2}}} \quad \text{(S3)}$$

We express $\mathrm{n(O)}_{\mathrm{TiO_2}}$ from equation (S3) and get

$$n(\mathrm{O})_{\mathrm{TiO_2}} = \frac{n(\mathrm{N})_\mathrm{b} - n(\mathrm{O})_\mathrm{b}(\mathrm{N/O})_\mathrm{a}}{(\mathrm{N/O})_\mathrm{a}} \quad \text{(S4)}$$

We calculate the molar fraction of oxygen and titanium coming from TiO_2 in the layer after degradation

$$x(\mathrm{Ti+O})_{\mathrm{TiO_2}} = \frac{n(\mathrm{O})_{\mathrm{TiO_2}} + n(\mathrm{Ti})_{\mathrm{TiO_2}}}{n(\mathrm{N})_\mathrm{b} + n(\mathrm{O})_\mathrm{b} + n(\mathrm{Ti})_\mathrm{b} + n(\mathrm{O})_{\mathrm{TiO_2}} + n(\mathrm{Ti})_{\mathrm{TiO_2}}} \quad \text{(S5)}$$

We can assume that the TiO_2 layer has the following titanium and oxygen ratio

$$n(\mathrm{Ti})_{\mathrm{TiO_2}} = \frac{1}{2} n(\mathrm{O})_{\mathrm{TiO_2}} \quad \text{(S6)}$$

$$x(\mathrm{Ti+O})_{\mathrm{TiO_2}} = \frac{\frac{3}{2}n(\mathrm{O})_{\mathrm{TiO_2}}}{n(\mathrm{N})_\mathrm{b} + n(\mathrm{O})_\mathrm{b} + n(\mathrm{Ti})_\mathrm{b} + \frac{3}{2}n(\mathrm{O})_{\mathrm{TiO_2}}} \tag{S7}$$

By assuming the same layer thickness of TiO_2 on both sides of the support, we can calculate the TiO_2 thickness as

$$\mathrm{thk}(\mathrm{TiO_2})_\mathrm{a} = \frac{\mathrm{thk(whole)_a}\,x(\mathrm{Ti+O})_{\mathrm{TiO_2}}}{2} \tag{S8}$$

Based on this, we can estimate the average thickness of the TiO_2 layer for the TiON sample to 3.0 ± 0.4 nm. The average thickness and standard deviation are calculated from all data points in **Figure 2** (after degradation data points). Note that the estimate of the linear regression extrapolation (2.8 nm) falls within the standard deviation of this method. Using the same estimation method on the TiON-Ir sample, we get an estimated average TiO_2 layer thickness of 0.3 ± 0.3 nm after EC-P and 0.0 ± 0.6 nm after EC-CV. The ten-fold difference between the estimated TiO_2 layer thickness for TiON and TiON-Ir sample suggests a ten times higher stability of the TiON support due to the presence of Ir. For the TiON-Ir sample, the increase of the average (N+O)/Ti ratio found in **Table S2** suggests that a TiO_2 layer has formed during both degradation protocols. Interestingly, for the TiON sample, the slight decrease of the average (N+O)/Ti ratio found in **Table S2** suggests that no TiO_2 layer has formed during the degradation protocol but instead the TiON structure remained and N atoms were exchanged for O atoms.

Table S2: Table of average elemental composition obtained from IL-EELS analysis for TiON, and TiON Ir samples. Values are written as mean ± standard deviation.

	TiON-Ir			TiON	
	Ir NPs	EC-P	EC-CV	BEFORE DEGRADATION	AFTER DEGRADATION
THICKNESS (nm)	6.9 ± 1.8	6.3 ± 1.3	6.5 ± 1.4	10.7 ± 2.5	11.2 ± 3.1
N	20.4 ± 2.7	17.8 ± 4.5	16.9 ± 5	31.1 ± 2.6	11.9 ± 4.1
Ti	48.4 ± 2.4	47.6 ± 4.3	47.4 ± 3.1	44.3 ± 2.5	45 ± 3.4
O	31.2 ± 3.2	34.6 ± 4.7	35.7 ± 5.1	24.6 ± 1.5	43 ± 3.8
N/O	0.67 ± 0.15	0.54 ± 0.2	0.5 ± 0.2	0.8 ± 0.11	0.28 ± 0.12
N/Ti	0.42 ± 0.06	0.38 ± 0.09	0.36 ± 0.11	0.71 ± 0.09	0.27 ± 0.1
O/Ti	0.65 ± 0.09	0.73 ± 0.14	0.76 ± 0.13	0.56 ± 0.05	0.96 ± 0.12
(N+O)/Ti	1.07 ± 0.1	1.11 ± 0.15	1.12 ± 0.12	1.26 ± 0.12	1.23 ± 0.16

S2. XPS characterization

XPS analyses were performed on Ti TEM grid samples after nitridation, Ir deposition, and electrochemical tests (EC-P and EC-CV). Information about the surface composition, oxidation states, and type of chemical bonding on the surface was obtained from the XPS data. The following peaks were detected in the XPS spectra: Ti 2p, Ti 3s, O 1s, N 1s, C 1s, K 2p, Si 2p, and Ir 4f. Surface composition considering elements O, Ti, N, and Ir after different stages for TiON-Ir was obtained and shown in **Table S3**. Carbon was assumed to be due to contamination, and it was not taken into account although it was detected. Also, elements Si and K were detected in the amount of a few at. %, but they were not considered for quantification. After nitridation, N is introduced into the TiO_2 structure resulting in 51 at. % of O, 23 at. % of N, and 26 at. % of Ti (approximately $Ti_1N_1O_2$). The excess O may be contributed to surface contamination, which is detected due to high surface sensitivity of the XPS method. High-energy resolution XPS spectra were acquired to get insight into the surface chemistry after different sample treatments. **Figure S3** shows XPS spectra Ti 2p and Ir 4f after Ir deposition, EC-P, and EC-CV for TiON-Ir sample. Ti 2p spectra consist of the Ti $2p_{3/2}$ and Ti $2p_{1/2}$ peaks separated by 5.9 eV. After nitridation, N 1s peak at 396.0 eV appears, related to the nitride or oxynitride formation. In addition to Ti $2p_{3/2}$ peak at 458.6 eV of Ti(4+) (50 %), two other peaks are at 456.0 eV (19 %) and 457.2 eV (31 %) are characteristic for Ti–(O, N) and Ti–N bonding, respectively.[1]

After the Ir deposition, about 1.6 ± 0.3 at. % of Ir is present on the surface. The XPS spectra of Ti 2p, O 1s, and N 1s do not change significantly after Ir-deposition concerning nitridated samples. The Ir 4f spectrum consists of an Ir $4f_{7/2}$ peak at 61.4

eV, and Ir $4f_{5/2}$ separated for 3.0 eV to the higher binding energy. After Ir deposition, the Ir $4f_{7/2}$ spectrum was deconvoluted in a peak at 61.4 eV related to the Ir(0) metallic state and the second peak at 62.6 eV, which is related to Ir-oxide in the Ir(4+) state.[2] Their intensities show that after Ir deposition, about 60 % of the Ir atoms are in a metallic state, and about 40 % are in Ir(4+). We should note that the Ir 4f spectrum overlaps with the Ti 3s spectrum at 60 eV, making it difficult to quantify Ir presence. After EC-P, the Ir 4f spectrum still shows the presence of two oxidation states of Ir, but the Ir(4+) state is 70 %, and Ir(0) presents 30 % of total Ir atoms (**Figure S3b**). After EC-CV, all Ir atoms were found in the Ir(4+) oxidation state (**Figure S3b**). The total concentration of Ir on TiON-Ir after EC-P is similar as after deposition, i.e., 1.5 ± 0.3 at. %, and after EC-CV, there is 1.0 ± 0.3 at. % of Ir. We observed that Ir concentration is decreased after EC-CV.

Another change observed by XPS after EC-P and EC-CV is that the concentration of N decreased, and O concentration increased (**Table S3**). This probably indicates the replacement of N with O during the catalytic reaction. The Ti 2p spectra after EC-P and EC-CV (**Figure S3e,f**) have similar features as a spectrum after nitridation, i.e., Ti(4+) peak at 458.6 eV and two peaks at 456.0 eV and 457.2 eV related with Ti–(O, N) and Ti–N bonds. However, after EC-P and EC-CV, the relative amount of TiO_2 increased from 50 % to 65 % at the expense of the Ti–(O, N) and Ti–N bonds, reflecting a decrease of total N concentration during EC-P and EC-CV.

N 1s spectra also undergo some changes (not shown here). In addition to the nitride peak at 396.0 eV, two peaks at 400.0 eV and 401.0 eV appeared after EC-P and EC-CV. They can be related to the transformation of the nitride bonds. They can be attributed to interstitial positions of N-atoms (peak at 400.0 eV) and the formation of N-O type species (peak at 401.0 eV) on the surface.

Table S3: The surface composition obtained by XPS in at. % of TiON-Ir, atomic ratios between elements, and portions of different Ir oxidation states in total Ir 4f XPS spectra.

	O	N	Ti	Ir	N/O	O/Ti	N/Ti	(N+O)/Ti	N/Ir	Ir(0)	Ir(4+)
	at. %									of total Ir	
**NITRIDATION**	51.2	22.7	26.1	0.0	0.44	1.9	0.87	2.8		0.0	0.0
**Ir DEPOSITION**	59.5	17.7	21.3	1.6	0.30	2.8	0.83	3.6	11.1	0.6	0.4
**EC-P**	68.8	13.1	16.6	1.5	0.19	4.1	0.79	4.9	8.7	0.3	0.7
**EC-CV**	67.5	14.1	17.4	1.0	0.21	3.9	0.81	4.7	14.1	0.0	1.0

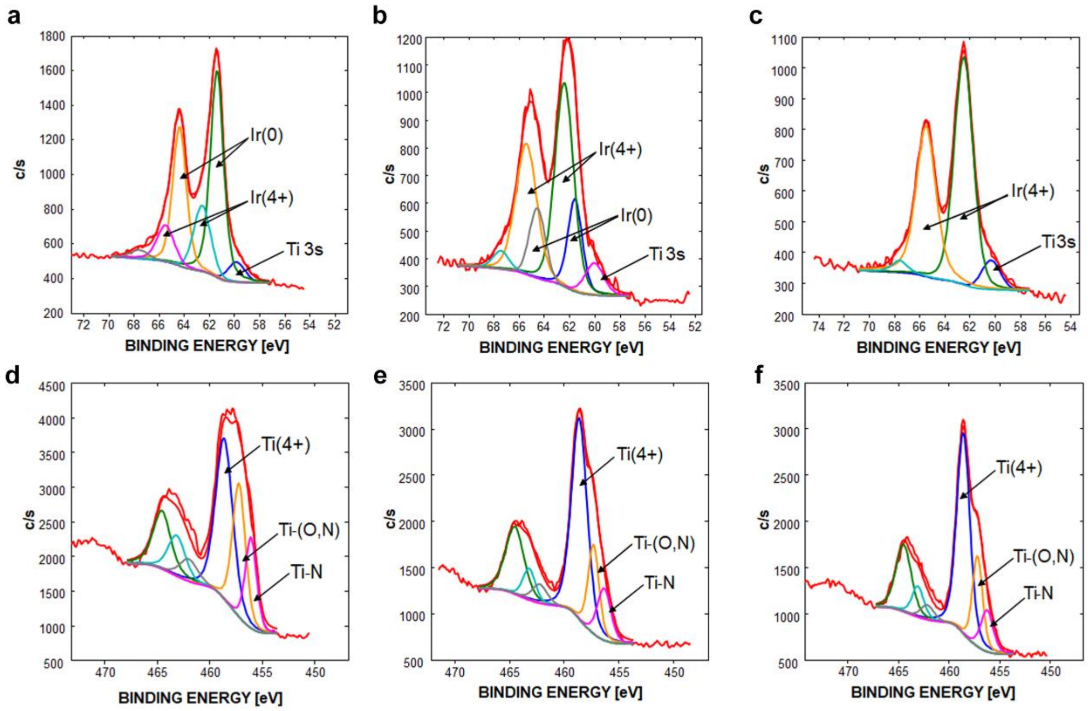

Figure S3: (**a, d**) XPS spectra of Ir 4f and Ti 2p after Ir deposition, (**b, e**) after EC-P, and (**c, f**) after EC-CV for sample TiON-Ir.

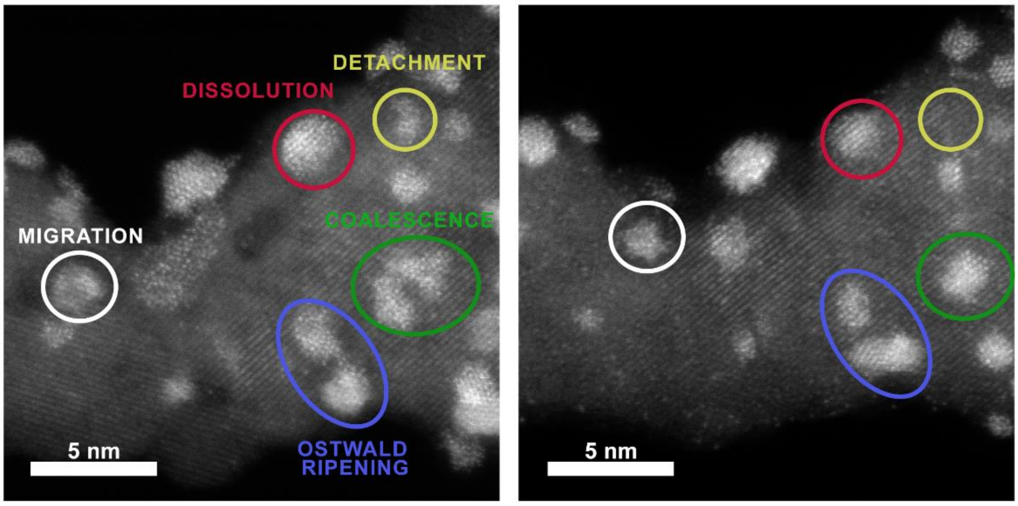

Figure S4: Identical location STEM-HAADF images of preliminary TiON-Ir sample before (**left**) and after (**right**) electrochemical degradation showing all the possible degradation processes in a single image pair.

S3. Raman spectroscopy

Raman spectroscopy was used as another technique to evaluate structural properties of TiON, TiON-Ir, after EC-P, and EC-CV samples. The characterization was performed in the ex-situ mode, i.e., before and after the electrochemical treatment (**Figures S5** and **S6)**. For each kind of sample, the spectra were measured on 3 different sites and, on each of them, by sequentially applying an increasing laser power. Such a measurement protocol enables estimations of the samples' stability and an example of such a measurement procedure is depicted in **Figure S5**. The inset clearly shows how the intensity in the as-measured spectra increases as a function of the increasing laser power. Of course, due to the high laser power some band changes can occur. It is thus self-evident that only Raman spectra recorded with the low laser powers can be used to interpret the structure. With the aim to enable comparison of the spectra, they were adapted to more similar intensities (**Figure S6**). The factors that were used to adapt the spectral intensities are not essential for a qualitative comparison. The Raman spectra of all measured sites are shown in **Figure S6** in the spectral range 20 cm^{-1} to 1100 cm^{-1}. Evidently, the spectra started to change gradually when the laser power of 3.4 mW was employed, but the extent of changes differed.

The spectra of the TiON sample (0.6 mW) reveal a broad group of bands in the investigated structural range (**Figure S6a** – green lines). These bands somewhat

resemble the shape of the bands characteristic for TiN: a doublet band at around 215 and 315 cm^{-1}, and a broad one between 540 cm^{-1} and 560 cm^{-1}.[3,4] It was reported that the positions of TiN bands could shift with changing stoichiometry.[5] Our spectra of the TiON samples (0.6 mW – green lines) show the bands at 203 cm^{-1}, 363 cm^{-1} and 506 cm^{-1} (sh), 579 cm^{-1} (**Figure S6a**), which are shifted with regard to TiN. We assume that the shifts are the consequence of the presence of Ti(4+)–O and Ti–O–N as shown by XPS. With increasing laser power (**Figure S6a**), the spectra almost retain their shape, with only some increase in the intensity of the bands at the lower wavenumbers with regard to the bands in the region above 540 cm^{-1} at the highest powers. No significant tendency to the formation of anatase was noted.

When TiON-Ir was examined, the spectra recorded using 0.6 mW (**Figure S6 B** – green spectra) showed similar bands together with the appearance of the characteristic 146 cm^{-1} band of anatase. With increasing laser power, the intensity of the sharp 146 cm^{-1} band increased dramatically on sites B1 and B2 with regard to the TiON sample without Ir particles (**Figure S6 A**). Obviously, a significant transformation to anatase occurred (**Figure S6 B**). The characteristic anatase triple bands also started to become visible. The B3 site, however, showed slower transformation.

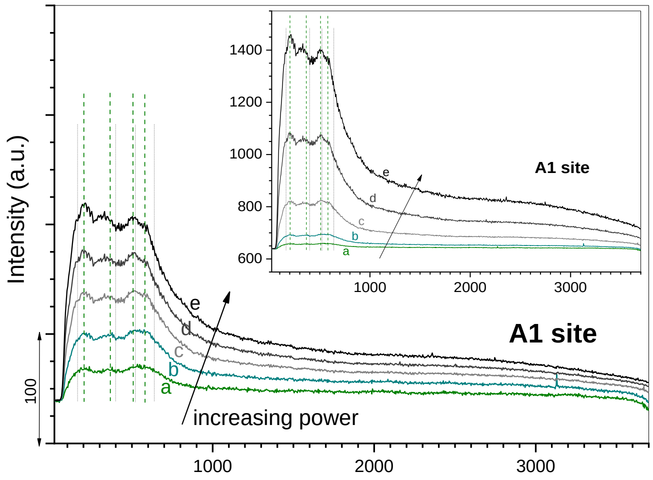

Figure S5: Raman spectra of TiON sample on the TEM grid. In inset, the spectra are depicted for the A1 site in the spectral range from 20 cm^{-1} to 3700 cm^{-1}. The spectra were measured sequentially using an increasing laser power of: a) 0.6 mW, b) 1.4 mW, c) 3.4 mW, d) 7.3 mW, and e) 13.5 mW. Arrows denote the direction of the increasing laser power. The spectra adapted for comparison are shown in the main graph. Green dashed lines denoted the $TiNO_x$ band while black dotted lines represent the positions of the anatase modes.

After EC-P: Interestingly, after the electrochemical treatment (**Figure S6 C**), the shape of the spectra recorded at 0.6 mW (green spectra) resembles the spectra of the TiON sample (**Figure S6 A**), with the bands appearing at 203 cm^{-1}, 363 cm^{-1}, and 515 cm^{-1} (sh), 579 cm^{-1}, marked with vertical green dashed lines. With increasing laser power, the stability of this sample is considerably improved with regard to the TiON-Ir sample (**Figure S6 B**).

After EC-CV: The spectra recorded after EC-CV revealed that these samples (**Figure S6 D**) were similarly stable as the TiON samples in **Figure S6 A**. Moreover, these spectra confirmed that two different types of sites exist. The spectra from the D1 site represent the N-rich site with bands more similar to the TiN spectrum,[3,5] the doublet band at 217 cm^{-1} and 321 cm^{-1}, and a broad band at 602 cm^{-1} with a shoulder at 529 cm^{-1}, marked with vertical solid red lines in **Figure S6 D**. The spectra from the other two sites (D2 and D3) resemble the spectra of the TiON samples (**Figure S6 A**). Evidently, the stability of EC–CV sample under the increasing laser power was comparable to that of the TiON samples.

Raman measurements clearly showed that the TiON and EC-CV samples were the most stable under the increasing laser power. The EC-P sample was somewhat less stable, while the TiON-Ir sample was more susceptible to transformation into anatase. The increased stability of the electrochemically treated EC-CV and EC-P samples during Raman measurement (**Figure S6 C,D**) supports our claim that the electrochemically formed Ir SAs are associated with the increased stability.

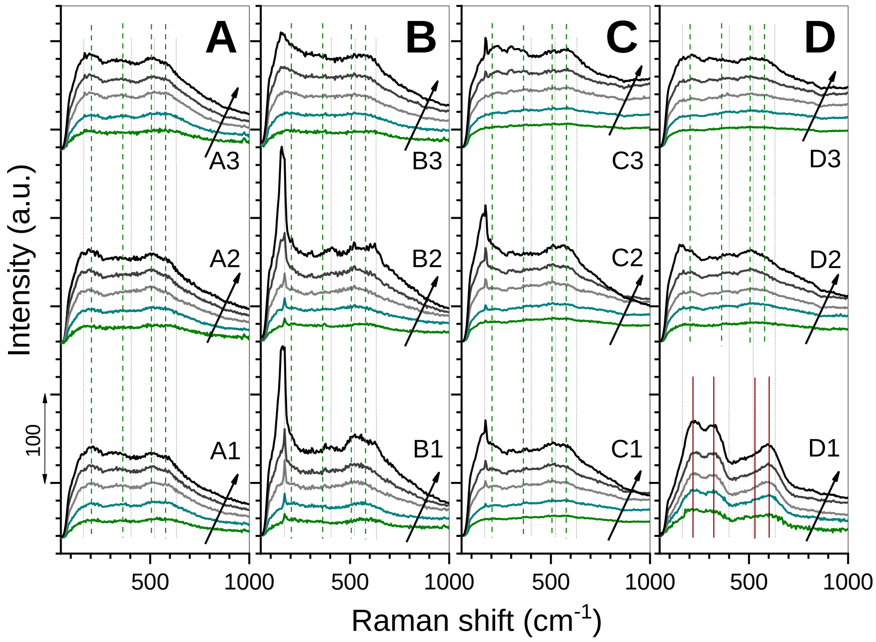

Figure S6: Raman spectra of samples on the TEM grid adapted for comparison: **A)** TiON, **B)** TiON-Ir, **C)** after EC–P, and **D)** after EC–CV. For each sample, the spectra are depicted for three sites in the spectral range from 20 cm^{-1} to 1100 cm^{-1}. The spectra were measured sequentially at each site using an increasing laser power of 0.6 mW, 1.4 mW, 3.4 mW, 7.3 mW, and 13.5 mW. Arrows denote the direction of the increasing laser power. Green dashed lines denoted the $TiNO_x$ band while black dotted lines represent the positions of the anatase modes.

S4. Iridium redox charge-normalized OER performance

The OER polarization curves obtained before/after sequential electrochemical treatments (i.e. *Before EC-P, After EC-P and After EC-CV*) were normalized also per characteristic Ir(III/IV) redox charge in the potential window of 0.6 -1.1 V, respectively. The charge was obtained during fast cyclic voltammetry in between treatments (**Figure S7a**). The most significant charge increase occurs after the EC-CV protocol (**Figure S7b**). Interestingly though are the following two facts. Firstly, the corresponding OER performance trend (**Figure S7c,d**) reveals a drop in performance with increasing the Ir surface area (in this case represented by the Ir charge) which is rather unusual since increasing the number of active sites should result in a constant specific OER activities, that is, if indeed all sites are equally active. Secondly, the increase of the surface area (i.e. Ir redox charge) partially (excluding the *After EC-P curve*) goes in line with the OER geometric current density trend (**Figure 1b,c**) where the highest current density is obtained after the EC-CV treatment (with the largest Ir charge). Overall, the two facts discussed above imply we cannot be certain of the

exact nature of the active sites. These seem to be governed by the type of electrochemical treatment and/or the extent of support Ir-interactions. The latter might as well be dictated by the type of electrochemical treatment.

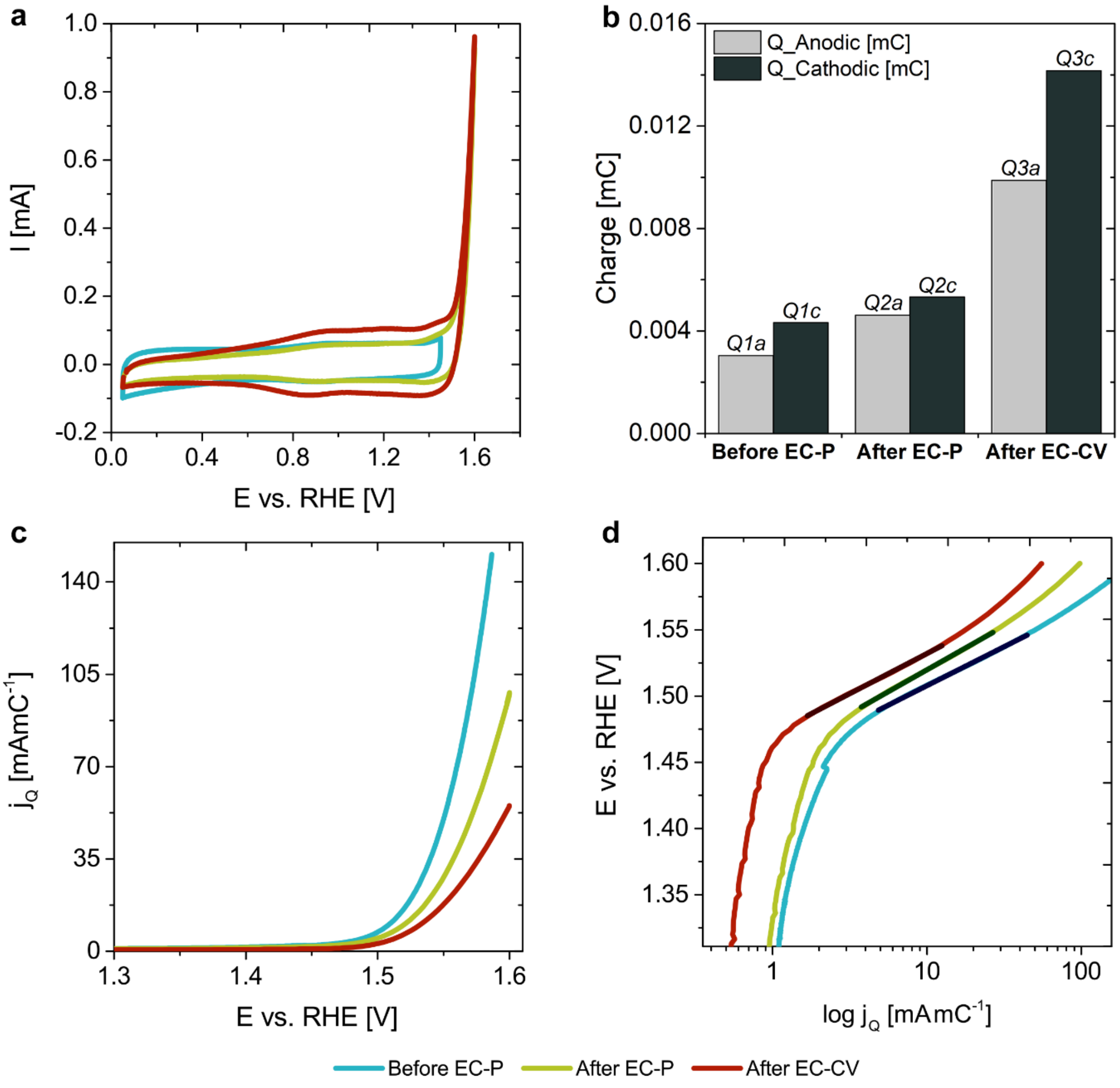

Figure S7: Electrochemical characterization of TiON-Ir TEM grid. **a)** Voltammetric response obtained under fast scan rate (300 mV s^{-1}). Either anodic or cathodic branch (0.6 – 1.1 V) was used to calculate the Ir charge. **b)** Iridium redox charge obtained from a) before or after EC-P and EC-CV electrochemical biasing, respectively. **c)** Iridium redox charge-normalized OER polarization curves measured either before or after EC-P and EC-CV electrochemical biasing, respectively. **d)** Tafel plots of OER polarization curves (constructed from c).

S5. Potentiostatic treatment of TiON, TiON-Ir and EELS analysis

A separate TiON-Ir sample (S-TiON-Ir) was prepared and electrochemically treated under identical conditions as TiON sample (1.6 V for 30 min, from here on refered to

as EC-stat). Before and after EC-stat an EELS analysis was performed, however under non-identical location mode (**Table S4**). The EELS results indicate that EC-stat induces similar trends as EC-P and EC-CV excluding the possibility of IL-EELS results being misinterpreted due to a non-identical electrochemical protocol. However, as evident from the corresponding electrochemical characterization, a static protocol did not enable sufficient removal of oxygen bubbles. More specifically, current response is gradually decreasing and eventually approaches bare TiON currents (**Figure S8**).

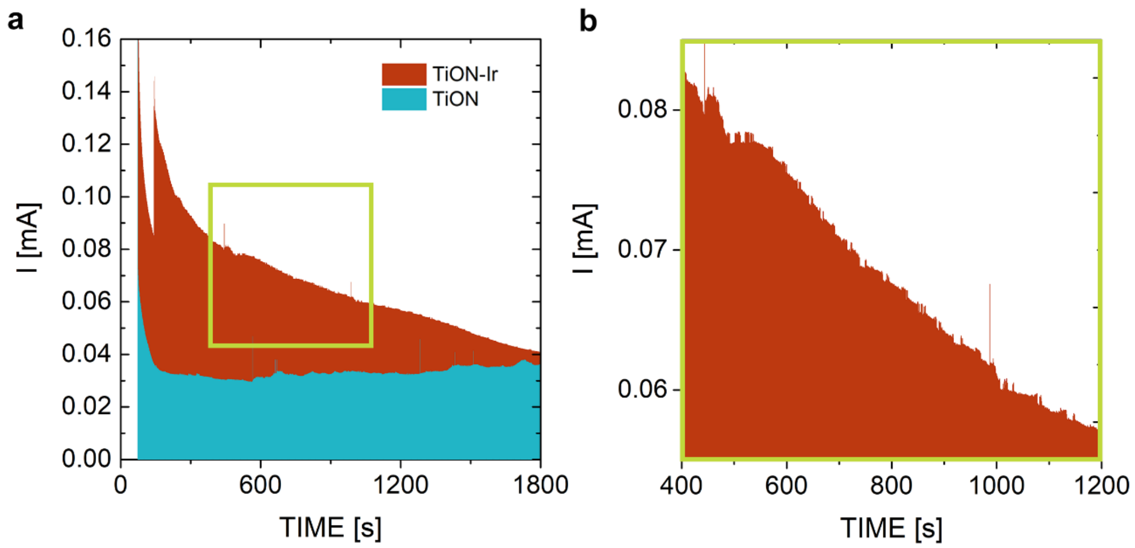

Figure S8: Electrochemical inspection of bubble management under vacuum suction. **a)** Current behaviour under chronoamperometry (1.6 V vs. RHE). **b)** Magnification of current response demonstrating small current oscillation due to bubble removal.

Table S4: Table of N/O ratio obtained from non-IL-EELS analysis for, S-TiON-Ir, and TiON-Ir samples. Values are written as mean ± standard deviation.

	**N/O ratio**		
	After EC-*stat*	After EC-P	After EC-CV
**S-TiON-Ir**	0.5 ± 0.09		
**TiON-Ir**		0.46 ± 0.09	0.42 ± 0.07

S6. DFT

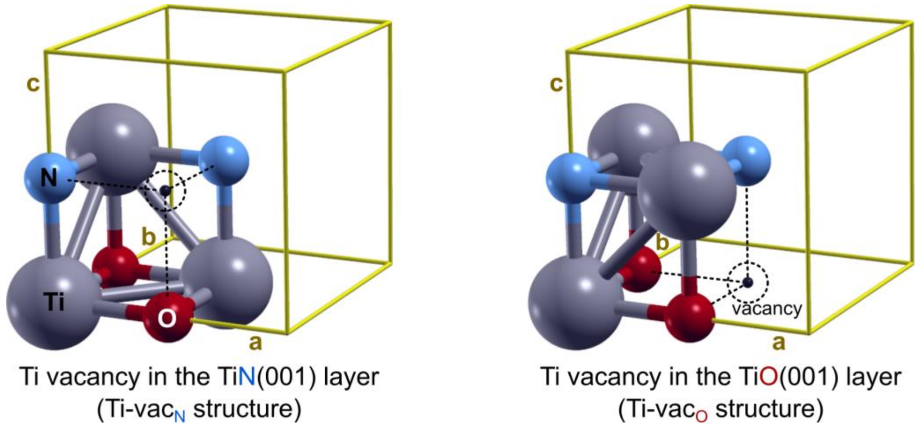

Figure S9: Cubic unit cells of a TiON bulk, containing 1 Ti-vacancy and 3 Ti, 2 O, and 2 N ions. The bulk structure consists of two interpenetrating fcc latices of Ti cations (with 25% of vacancies) and O/N anions, shifted by half a Bravais lattice vector with respect to one another. Ti-vacancy can be located in either the TiN(001) or TiO(001) layer; the resulting "ordered" structures are named as Ti-vac$_N$ and Ti-vac$_O$, respectively, where the N and O subscripts indicate in which layer Ti-vacancies are located.

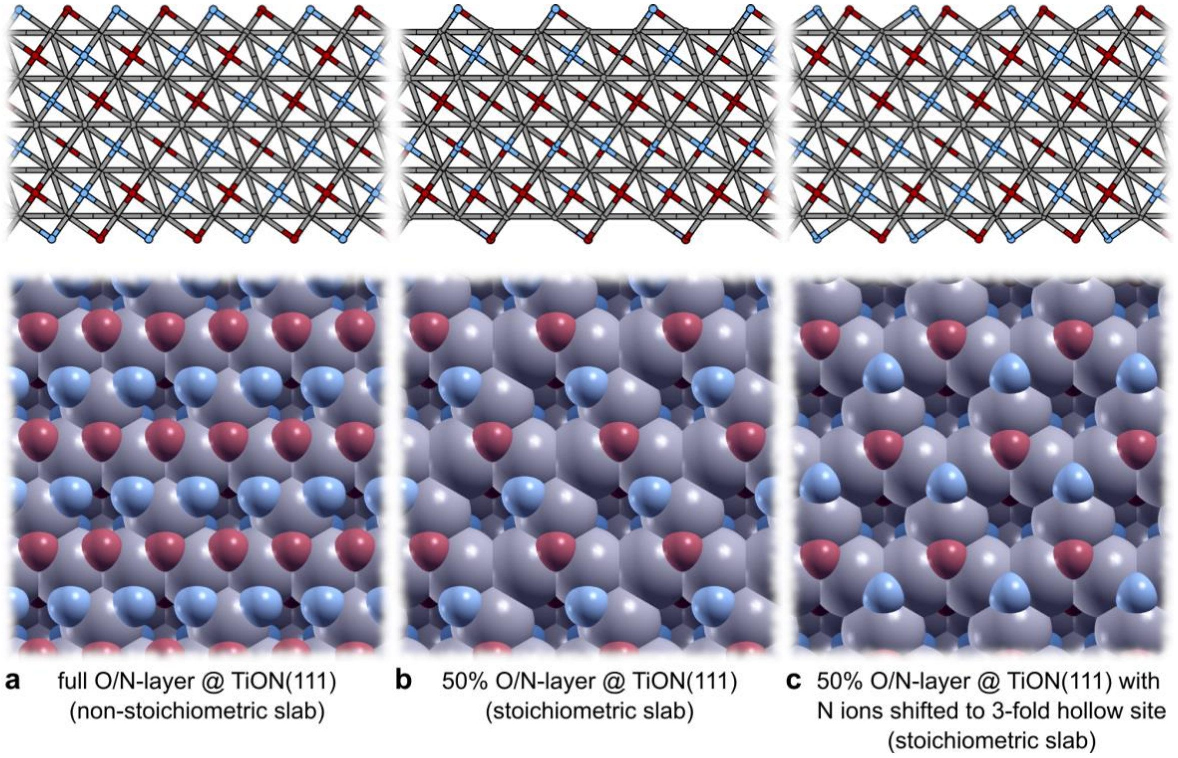

Figure S10: Sideview (top) and topview (bottom) snapshots of the utilized TiON(111) slabs that are five Ti-layers thick; only Ti-vac$_N$ structures are shown. (a) A non-stoichiometric slab terminated with the complete O/N layer on both sides. (b) A stoichiometric slab terminated on both sides with the O/N layer containing only 50 % of O and N ions. (c) Similar to (b), but N ions are shifted from vacancy edges to 3-fold hollow sites.

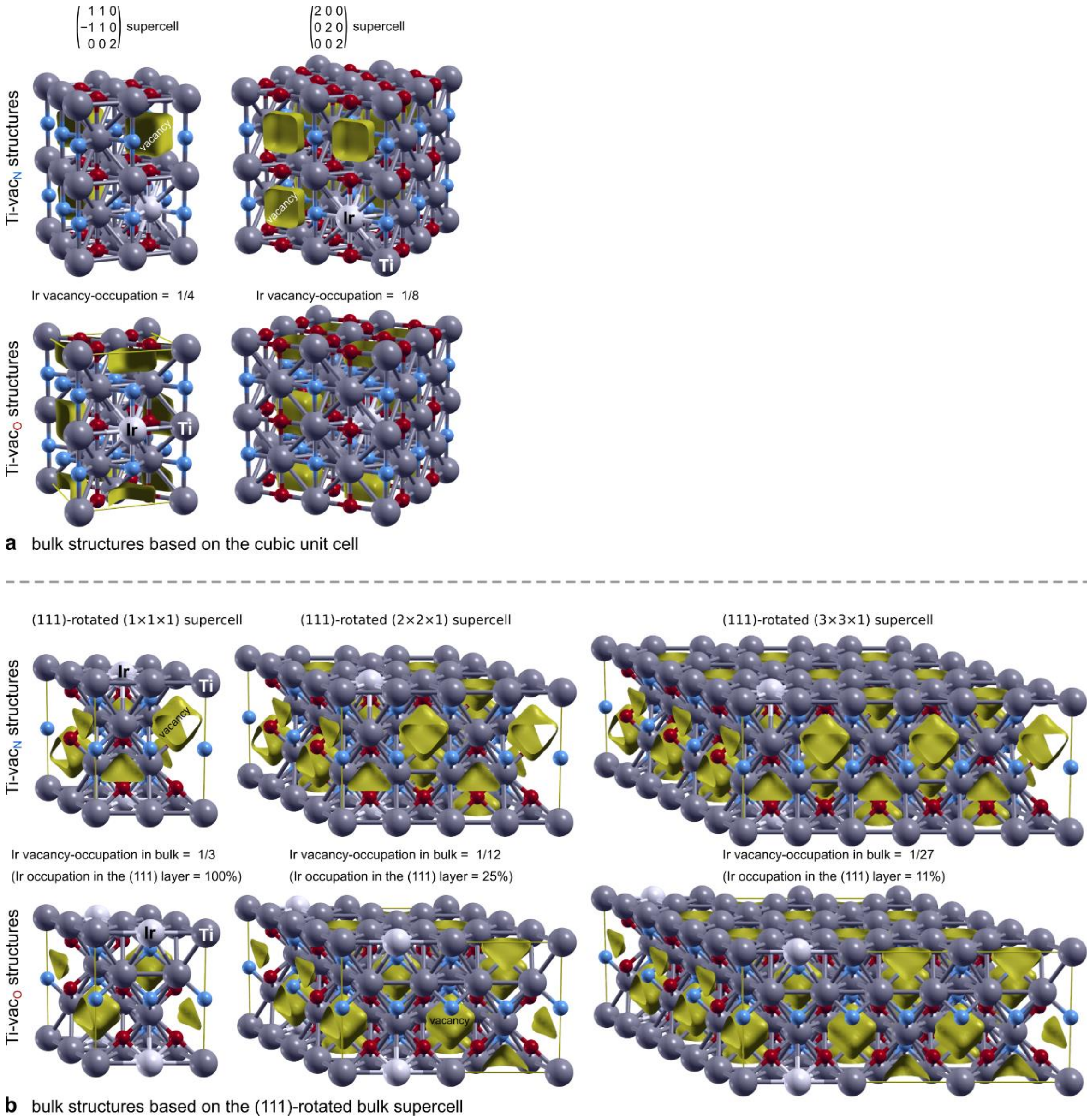

Figure S11: Snapshots of bulk TiON supercells, used to calculate the Ir induced N preference, $\Delta_{1:1}$ of eq (4) in the main article. (a) Supercells based on the cubic unit cell, and (b) supercells based on the (111)-rotated bulk cell. Both Ti-vac$_N$ and Ti-vac$_O$ structures are shown. There is always one Ir atom per supercell (in cases where more than one Ir atom appears, the additional Ir atoms are periodic replicas); the corresponding Ir vacancy-occupations are stated for each supercell. The yellow isosurfaces indicate Ti vacancies.

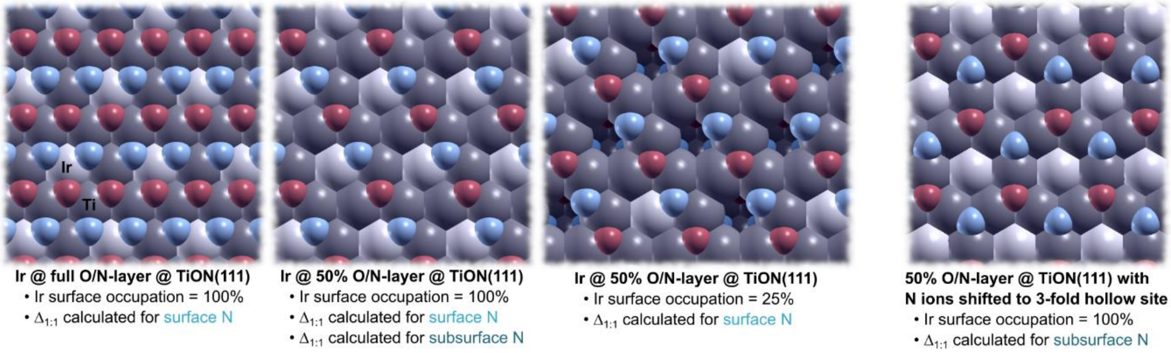

Figure S12: Topview of single atom Ir @ TiON(111) structures used to calculate the Ir induced N preference, $\Delta_{1:1}$ of eq (4) in the main article; only Ti-vac$_N$ structures were considered. Below each snapshot, the Ir vacancy-occupation in the surface Ti-layer is stated. The $\Delta_{1:1}$ values were calculated either for surface or subsurface N atoms. For the rightmost structure, $\Delta_{1:1}$ was calculated only for subsurface N atoms because surface N atoms do not bond to Ir atoms.

References

(1) Han, J. H.; Bang, J. H. A Hollow Titanium Oxynitride Nanorod Array as an Electrode Substrate Prepared by the Hot Ammonia-Induced Kirkendall Effect. *J. Mater. Chem. A* **2014**, *2* (27), 10568–10576. https://doi.org/10.1039/c4ta01469c.

(2) Pfeifer, V.; Jones, T. E.; Velasco Vélez, J. J.; Massué, C.; Arrigo, R.; Teschner, D.; Girgsdies, F.; Scherzer, M.; Greiner, M. T.; Allan, J.; Hashagen, M.; Weinberg, G.; Piccinin, S.; Hävecker, M.; Knop-Gericke, A.; Schlögl, R. The Electronic Structure of Iridium and Its Oxides. *Surf. Interface Anal.* **2016**, *48* (5), 261–273. https://doi.org/10.1002/sia.5895.

(3) Ding, Z. H.; Yao, B.; Qiu, L. X.; Lv, T. Q. Raman Scattering Investigation of Nanocrystalline δ-TiNx Synthesized by Solid-State Reaction. *J. Alloys Compd.* **2006**, *421* (1–2), 247–251. https://doi.org/10.1016/j.jallcom.2005.11.017.

(4) Doiron, B.; Li, Y.; Mihai, A.; Bower, R.; Alford, N. M. N.; Petrov, P. K.; Maier, S. A.; Oulton, R. F. Plasmon-Enhanced Electron Harvesting in Robust Titanium Nitride Nanostructures. *J. Phys. Chem. C* **2019**, *123* (30), 18521–18527. https://pubs.acs.org/doi/10.1021/acs.jpcc.9b03184.

(5) Spengler, W.; Kaiser, R.; Christensen, A. N.; Müller-Vogt, G. Raman Scattering, Superconductivity, and Phonon Density of States of Stoichiometric and Nonstoichiometric TiN. *Phys. Rev. B* **1978**, *17* (3), 1095–1101. https://doi.org/10.1103/PhysRevB.17.1095.
